# The Protease Activated Receptor2 Promotes Rab5a Mediated Generation of Pro-metastatic Microvesicles

**DOI:** 10.1038/s41598-018-25725-w

**Published:** 2018-05-09

**Authors:** Kaushik Das, Ramesh Prasad, Sreetama Roy, Ashis Mukherjee, Prosenjit Sen

**Affiliations:** 1 Department of Biological Chemistry, Indian Association for the Cultivation of Science, Kolkata, 700032 India; 2 Netaji Subhash Chandra Bose Cancer Research Institute, Kolkata, 700016 India

## Abstract

Metastasis, the hallmark of cancer propagation is attributed by the modification of phenotypic/functional behavior of cells to break attachment and migrate to distant body parts. Cancer cell-secreted microvesicles (MVs) contribute immensely in disease propagation. These nano-vesicles, generated from plasma membrane outward budding are taken up by nearby healthy cells thereby inducing phenotypic alterations in those recipient cells. Protease activated receptor 2 (PAR2), activated by trypsin, also contributes to cancer progression by increasing metastasis, angiogenesis etc. Here, we report that PAR2 activation promotes pro-metastatic MVs generation from human breast cancer cell line, MDA-MB-231. Rab5a, located at the plasma membrane plays vital roles in MVs biogenesis. We show that PAR2 stimulation promotes AKT phosphorylation which activates Rab5a by converting inactive Rab5a-GDP to active Rab5a-GTP. Active Rab5a polymerizes actin which critically regulates MVs shedding. Not only MVs generation, has this Rab5a activation also promoted cell migration and invasion. We reveal that Rab5a is over-expressed in human breast tumor specimen and contributes MVs generation in those patients. The involvement of p38 MAPK in MVs-induced cell metastasis has also been highlighted in the present study. Blockade of Rab5a activation can be a potential therapeutic approach to restrict MVs shedding and associated breast cancer metastasis.

## Introduction

Earlier microvesicles (MVs) were considered as pro-coagulant “dust” which were generated from activated human blood platelets^1^. Recently, they came up as an important contributor in intercellular communication along with direct cell-cell contact and cellular secretary molecules. These nano-sized vesicles (100–1000 nm in diameter), also termed as “ectosomes” or “microparticles: MPs”, released from almost all types of cells and basically formed by outward budding of plasma membrane. Unlike MVs, exosomes are small membranous entities of endocytic origin (30–80 nm in diameter) produced inside the multivesicular bodies (MVBs) and are released into the surrounding microenvironment following MVBs fusion with plasma membrane^2^. MVs transport genetic information in the form of mRNA, miRNA and bioactive protein molecules between cells^3–6^. They are abundant in the body fluids^7,8^ of patients suffering from diabetes; atherosclerosis, liver disease, kidney and cardiovascular disease or cancer and contribute to the progression of the disease^9–16^. Cancer cell-secreted MVs readily fuse with nearby healthy cells and act as a potent inducer of cellular transformations via the up regulation of cell migration, invasion and angiogenesis^17^.

Rabs are categorized as Ras oncogene family of monomeric G-proteins primarily involved in intracellular transport of small membranous vesicles. Rabs are located in the cytoplasm and linked with the vesicles via lipid tethers. They comprise of two forms, an active GTP bound form and an inactive GDP bound state^18^. Different Rabs are dedicated to perform various different functions inside the cells. Rab5a are located predominantly at the plasma membrane and are mainly associated with endocytosis^19^. Recent evidences suggest that oncogenic Rab5a is over-expressed in human breast carcinoma tissues and plays vital roles in the disease progression^20^, although the underlying mechanism is yet to be explored. Over-expression of Rab5a is also associated with enhanced motility of human muscle cells by altering cellular actin dynamics.

The contribution of protease activated receptor 2 (PAR2) in human breast cancer progression has been well established^21^, although its role in cancer propagation has not been focused yet. Previous reports document the direct involvement of PAR2 in cancer cell proliferation, metastasis and angiogenesis although the underlying mechanism is yet unraveled^22,23^. PAR2 cleavage by trypsin leads to the intracellular activation of ERK^1^/_2_ and AKT which performs various functions in cancer cells^24^.

The role of trypsin dependent PAR2 activation in pro-metastatic MVs generation from human breast cancer cells has not been studied although its role in breast cancer progression is well-established^21^. The involvement of AKT in PAR2-triggered MVs generation remains to be explored. Although AKT driven Rab5a activation is well reported^25^, the role of Rab5a in the context of PAR2-mediated MVs generation has not been identified. Furthermore, the contributions of these PAR2-derived MVs in promoting breast cancer migration and invasion as well as its underlying mechanism have not been well-established. In the present study, we have specifically investigated the mechanism of MVs generation from PAR2-activated human breast cancer cells and the consequences of MVs shedding in the propagation of the disease.

## Results

### Trypsin triggers MVs generation from human breast cancer cells via Rab5a activation

**Figure 1:**
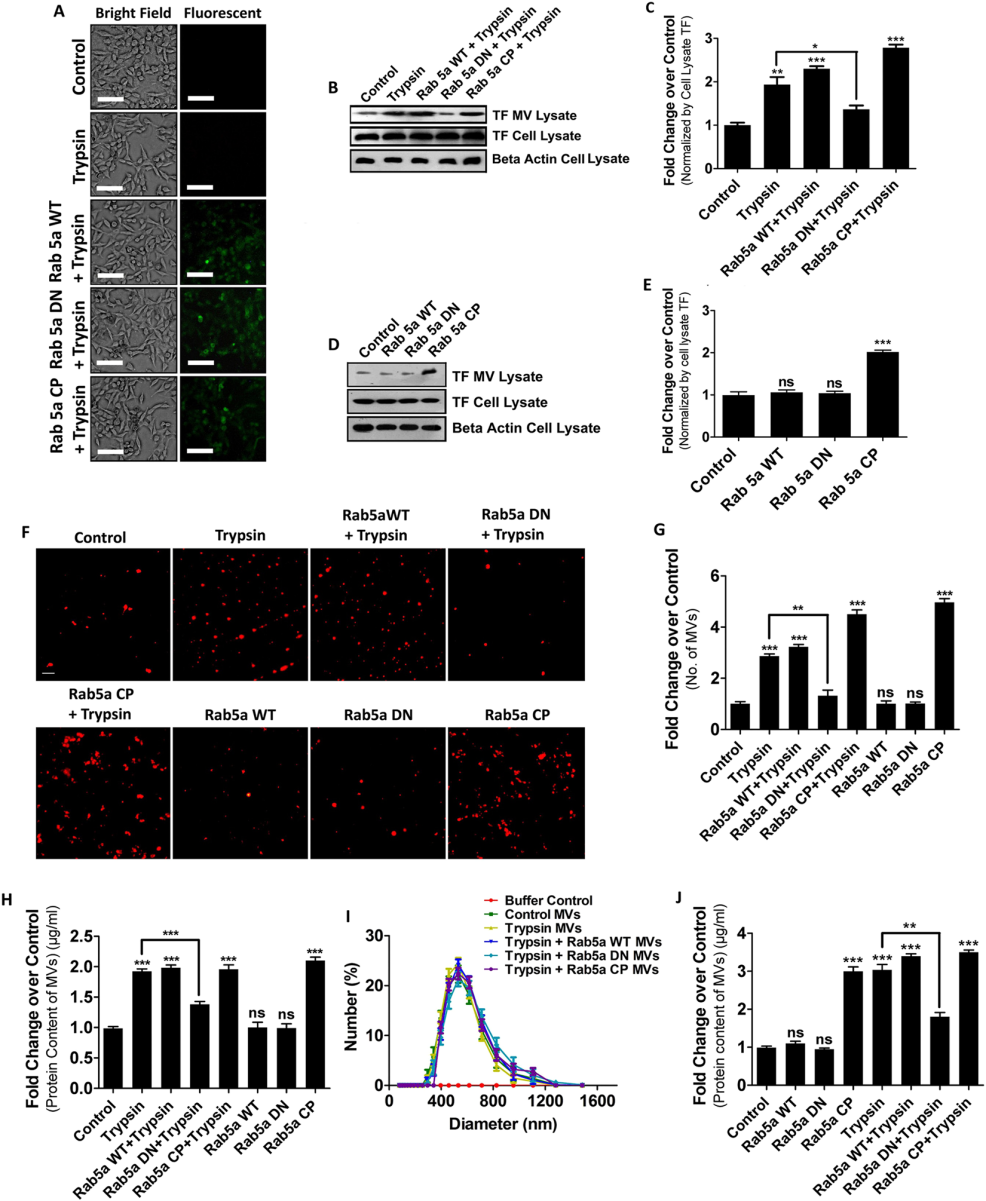
Trypsin Induces Microvesicles Generation from Human Breast Cancer Cells via Rab5a Activation. MDA-MB-231 cells were transfected with GFP-tagged Rab5a WT or Rab5a DN or Rab5a CP with the help of Lipofectamine 2000 (**A**). 48 hours later, cells were serum starved for 2 hours followed by treatment with trypsin (5 nM). 24 hours later, MVs were collected from the cell-supernatant and quantified by western blotting with MVs marker protein, TF and accordingly graph was prepared by GraphPad Prism5 (**B**) and (**C**). MDA-MB-231 cells were transfected with Rab5a constructs alone without trypsin addition and MVs quantification was done by western blotting (**D**) and (**E**). MVs were isolated, stained with a lipophilic dye, Nile Red and imaged by a fluorescent microscope. From the images MVs numbers were quantified and graph was generated by GraphPad Prism5 (**F**) and (**G**). MVs were lysed with lysis buffer and protein concentration was measured by Bradford assay and accordingly a comparative graphical analysis was performed by GraphPad Prism5 (**H**). MVs were suspended in 1X PBS followed by size analysis by DLS (**I**). MCF-7 cells were transfected with plasmid constructs of various Rab5a (WT, DN and CP) alongside control. After 48 hours, cells were challenged with trypsin overnight followed by MVs isolation as described in Methods. MVs were quantified by protein content analysis by Bradford assay and accordingly comparative graphical analysis was performed (**J**). Data presented over here are as Mean +/− S.E. of the Mean and differences are statistically significant at p < 0.05 using students’t-test after repeating the experiments at least thrice.

Previous studies have demonstrated that some highly metastatic human breast cancer cell lines (as MDA-MB-231) are capable of releasing large number of MVs into the surrounding environment^26^. The role of MVs in promoting tumor metastasis is well documented^17^ and reports suggest that PAR2 activation during some patho-physiological condition directly promotes cancer metastasis^27^. PAR2 is ubiquitously expressed in MDA-MB-231 cell lines^28^. Interestingly, high to moderate expression of Rab5a is observed in both MDA-MB-231 cell lines as well as human breast cancer tissues^20^ and knock-down of Rab5a significantly lowers cervical cancer cell motility^29^. Hence, we are trying to elucidate whether PAR2 activation by trypsin^30^ contributes to MVs generation from MDA-MB-231 cell lines and also to decipher the involvement of Rab5a in that process. For this purpose, we had transiently expressed wild-type Rab5a, its inactive dominant negative mutant Rab5a DN and active constitutive positive form Rab5a CP in MDA-MB-231 cells (Fig. 1A) followed by the treatment of trypsin along with untreated controls. MVs were isolated from the cell-supernatant and quantified by western blotting with a MVs marker protein, tissue factor (TF). The data suggest that trypsin treatment significantly increased MVs population in the cell-supernatant except for the Rab5a DN pre-transfected cells where no distinct changes in MVs count were observed (Fig. 1B,C). Interestingly, Rab5a CP alone transfected cells were capable of releasing more such MVs without trypsin addition whereas transfection with Rab5a DN or Rab5a WT failed to do so suggesting that Rab5a activation is mandatory in trypsin induced MVs generation (Fig. 1D,E). Cellular TF expression did not alter much after over-expressing Rab5a constructs followed by trypsin treatment. Also, trypsin addition did not interfere with endogenous Rab5a expression (Supplementary Fig. S1A,B). Next, MVs were collected from the cell-supernatant after various treatments and stained with a lipophilic dye Nile Red. Nile Red stained MVs were imaged directly by a fluorescent microscope from which MVs number was quantified by using Image J (Fig. 1F,G). Again, MVs were purified and total MVs protein content was estimated by Bradford assay (Fig. 1H). In consistent with our western blot data, both Nile Red staining approach and MVs protein estimation suggest that trypsin induces MVs production from MDA-MB-231 cells via the activation of Rab5a. Here also, Rab5a CP transfection into the cells alone was sufficient to generate more MVs, again confirming the mandatory requirement of Rab5a activation in MVs biogenesis. DLS analysis suggested that all the MVs were ranging in 300–1200 in diameter (Fig. 1I). To confirm this further, we implemented the same experiment in another human breast cancer cell line MCF-7 (constitutively expressing PAR2) where we over-expressed all forms of Rab5a (WT, DN and CP) followed by the addition of trypsin and quantified MVs generation by MVs protein estimation by Bradford assay (Fig. 1J). As consistent with our MDA-MB-231 data, here also we observed that activation of Rab5a plays vital roles in trypsin induced MVs generation.

Overall, the data in Fig. 1 clearly depict that trypsin stimulates MVs generation from human breast carcinoma cells that involves the activation of Rab5a from inactive GDP-bound state to an active GTP-bound form.

### Trypsin mediated MVs generation from MDA-MB-231 cells happens through PAR2 receptor activation

**Figure 2:**
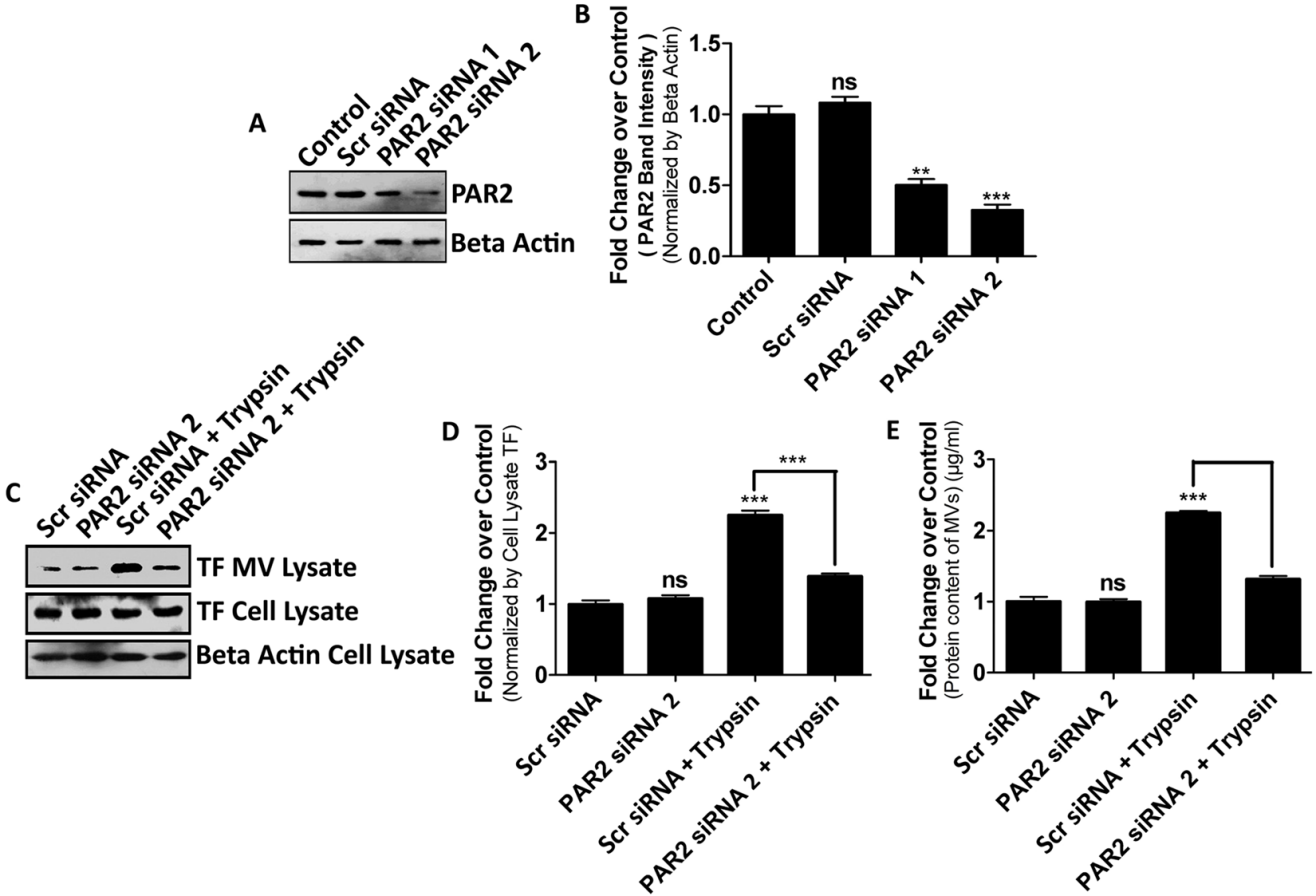
Trypsin-mediated Microvesicles Generation from MDA-MB-231 Cells Is Happening Through PAR2 Receptor Activation. MDA-MB-231 cells were transfected with 100 nM concentration of two different siRNAs against PAR2 alongside a scrambled control. 48 hours later, cells were lysed and PAR2 knock-down was analyzed by western blotting with PAR2 specific antibody and quantification was made by GraphPad Prism5 (**A**) and (**B**). Cells were transfected with PAR2 siRNA 2 followed by the addition of trypsin. 24 hours later, MVs quantification was done by western blotting with TF marker (**C**) and (**D**). MVs quantification was also performed by Bradford assay (**E**). Data presented here are as Mean +/− S.E. of the Mean. Differences are considered to be statistically significant at p < 0.05 using student’s t-test after performing the experiments three times whereas ‘ns’ represents non-significant changes.

Trypsin is a serine protease well known for activating PAR2, a GPCR family protein, via cleavage at N-terminal extracellular domain^31^. We were curious whether trypsin mediated MVs generation from MDA-MB-231 cells is happening through PAR2 receptor activation or not. To test this, we monitored impacts of siRNA mediated depletion of PAR2 on MVs generation. Of the two siRNAs tested (Fig. 2A,B), PAR2 siRNA 2 worked better and thus, was selected for subsequent experiments. We treated the cells with PAR2 siRNA2 prior to the addition of trypsin alongside an untreated control and quantified subsequent MVs generation both by western blotting and Bradford assay (Fig. 2C–E). Knock-down of PAR2 dramatically reduced MVs count despite trypsin addition which suggests that trypsin mediates MVs generation at least in part via activation of the PAR2 receptor.

### Trypsin induced Rab5a activation promotes MVs generation through actin polymerization

**Figure 3:**
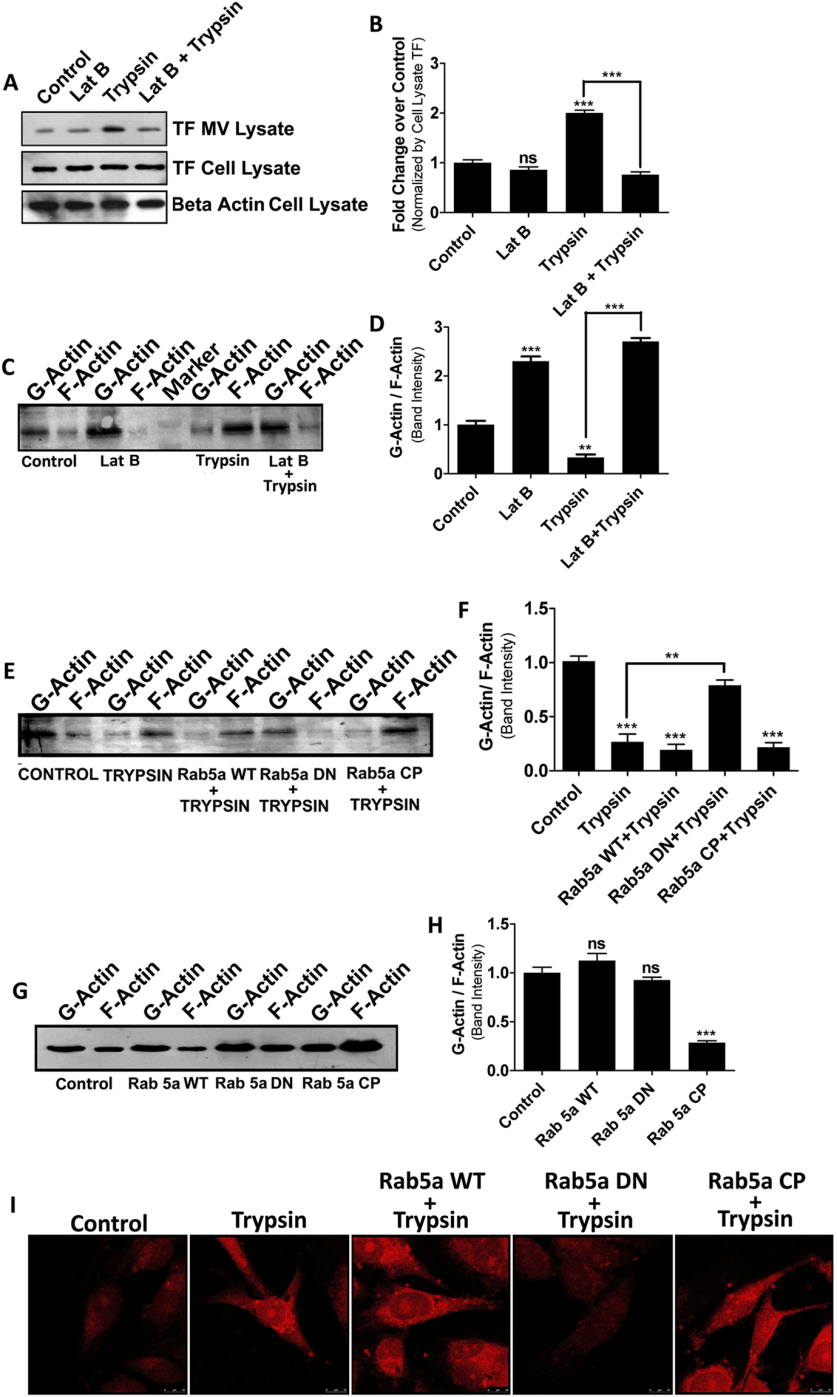
Trypsin Induced Rab5a Activation Promotes MVs Generation via Polymerization of Actin. MDA-MB-231 cells were challenged with Latrunculin B (200 µM) for an hour followed by treatment with trypsin and MVs generation was analyzed by western blotting with TF antibody and graph was prepared by GraphPad Prism5 (**A**) and (**B**). G-/F-actin quantification was also performed of these cells to determine the extent of actin polymerization (**C**) and (**D**). Cells were transfected with plasmid constructs of Rab5a, followed by trypsin treatment and G-/F-actin quantification was analyzed (**E**) and (**F**). Cells were transfected with various Rab5a constructs and G-/F-actin ratio was measured accordingly (**G**) and (**H**). MDA-MB-231 cells were challenged with Rab5a plasmids followed by trypsin addition. 5 hours later, cells were fixed with 4% para-Formaldehyde, permeabilized with 0.1% Triton-X-100 and stained with phalloidin red. Images were taken by a confocal microscope and the extent of actin polymerization was monitored. Data presented over here are as Mean +/− S.E. of the Mean and differences are statistically significant at p < 0.05 using student’s t-test. The experiments were performed for at least three times.

Previous studies have demonstrated that Rab5a activation is required to form invadopodia from cell surface^32^ for cell migration which also demands the dynamic role of actin^33^. MVs budding from cell surface are also accompanied by the polymerization of actin cytoskeleton^34^. Experiments presented in Fig. 1 demonstrate that Rab5a activation is pre-requisite for trypsin mediated MVs release from MDA-MB-231 cells. Hence, we questioned whether Rab5a activation promotes the polymerization of actin which might be critical for trypsin mediated MVs generation from cell surface. First of all, we pre-treated the cells with Latrunculin B, which retards the polymerization of actin and quantified MVs by western blotting with MVs marker TF upon trypsin addition (Fig. 3A,B). Result suggests that trypsin mediated MVs generation dramatically lowered upon perturbation of actin polymerization. As a measure of actin polymerization, we quantified G/F-actin ratio of Latrunculin B pre-treated trypsin induced cells alongside control (Fig. 3C,D). The data manifest that G/F-actin ratio was declined upon the administration of trypsin suggesting that actin polymerization was induced by trypsin which was reversed when Latrunculin B was incorporated before trypsin treatment. Next, we investigated whether Rab5a activation promotes actin polymerization or not which is essential in MVs biogenesis. G/F-actin quantification data suggest that trypsin induced Rab5a activation causes the polymerization of actin but when inactive form of Rab5a (Rab5a DN) was introduced into the cells no significant polymerization of actin results despite trypsin treatment (Fig. 3E,F). This indicates the requirement of active Rab5a in trypsin induced actin polymerization which might regulate MVs generation. Rab5a CP transfection alone can induce actin polymerization without trypsin treatment also confirming the involvement of active Rab5a in actin polymerization based MVs shedding (Fig. 3G,H). Again, to confirm Rab5a activation related actin polymerization, we transfected the cells with various forms of Rab5a followed by trypsin addition and stained the cells with phalloidin-red (a dye that typically binds to the filamentous form of actin) (Fig. 3I). Confocal microscopic analysis clearly demonstrates that actin polymerization has taken place in Rab5a activated cells. To confirm this further, we transfected MDA-MB-231 cells with Rab5a siRNA (Supplementary Fig. S1C) and analyzed both MVs count (Supplementary Fig. S1D,E) as well as G/F actin ratio (Supplementary Fig. S1F,G) upon trypsin challenge by western blotting. The data also suggest that knock-down of Rab5a not only impairs trypsin induced actin polymerization but also affects MVs generation from the cells.

Finally, we examined the role of PAR2 in trypsin induced Rab5a activation and actin polymerization related to MVs generation. We transfected MDA-MB-231 cells with PAR2 siRNA and measured G/F actin ratio after trypsin challenge. The results suggest that PAR2 knock-down does not alter endogenous Rab5a expression (Supplementary Fig. 2A,B) rather decreases trypsin induced actin polymerization (Supplementary Fig. 2C,D). This implies that PAR2 is essential in trypsin mediated polymerization of actin which ultimately regulates MVs shedding. In order to determine the effect of Rab5a over-expression (WT, DN and CP) on MVs generation in PAR2 depleted cells, we transfected all three forms of Rab5a gene into PAR2 knocked-down cells and analyzed the generation of MVs by western blotting (Supplementary Fig. 2E). The data suggest that in PAR2 knocked-down cells trypsin treatment does not induce MVs generation despite over-expression of Rab5a WT or inactive Rab5a DN. But active Rab5a CP over-expression is sufficient to induce MVs generation in PAR2 depleted cells irrespective of trypsin addition. This suggests that the activation of Rab5a is PAR2 dependent which is essential in MVs biogenesis.

As a whole, all these observations emphasize that trypsin mediated PAR2 stimulation promotes actin polymerization via the activation of Rab5a, crucial for MVs shedding from the cell surface.

### Rab5a activation by trypsin leading to MVs generation occurs via intracellular activation of AKT

**Figure 4:**
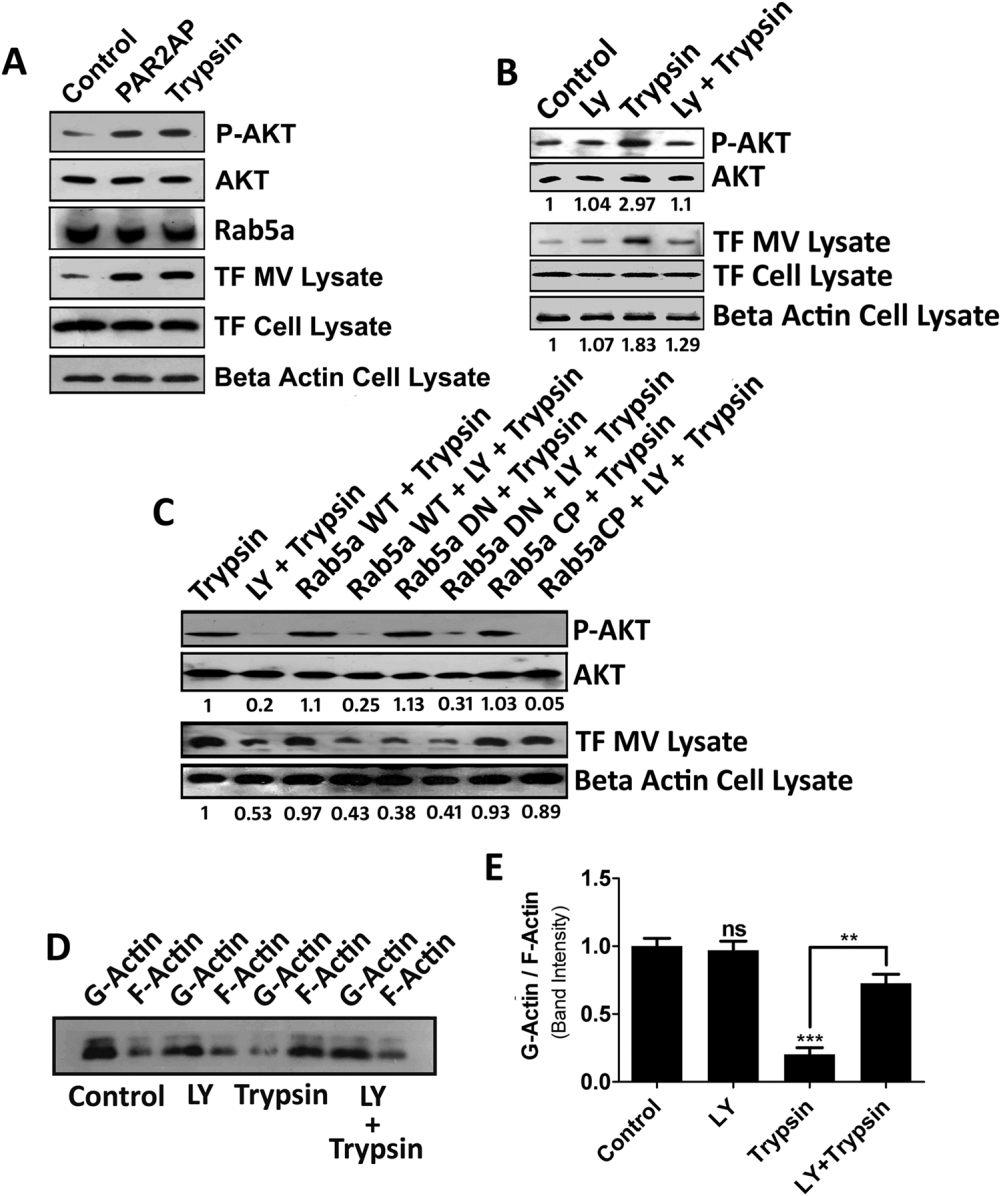
Trypsin Mediated Rab5a Activation Leading to MVs Generation Occurs via Intracellular Activation of AKT. Cells were treated with PAR2AP (100 µM) and trypsin followed by an incubation of 10 minutes and the phosphorylation of AKT was analyzed by western blotting. Subsequent MVs generation was quantified 24 hours later by western blotting with MVs marker, TF (**A**). Cells were serum starved for an hour followed by treatment with LY294002 (25 µM). An hour later trypsin was added and both AKT phosphorylation as well as MVs generation was quantified by western blotting (**B**). Cells were transfected with various forms of Rab5a followed by LY294002 and trypsin addition. Both AKT phosphorylation as well as MVs generation was estimated by western blotting to assess the chronology of activation (**C**). Cells were challenged with LY294002 followed by trypsin treatment and G-/F-actin ratio was quantified to determine the effect of AKT activation on actin polymerization (**D**) and (**E**). Data presented over here are as Mean +/− S.E. of the Mean and differences are statistically significant at p < 0.05 using student’s t-test after performing the experiments thrice.

Next, we focused on the signaling events essential for trypsin mediated MVs generation from MDA-MB-231 cells. Previous studies have demonstrated that PAR2 cleavage results in PI3K-dependent AKT activation which plays vital roles in cancerous progressions^35,36^. AKT activation also contributes to Rab5a activation^25^. Therefore, we investigated whether trypsin mediated activation of AKT plays any part in MVs generation from MDA-MB-231 cells and also to correlate AKT and Rab5a activation in MVs biogenesis. Treatment of cells with trypsin alongside PAR2 activation peptide (PAR2AP; positive control) not only resulted in AKT activation via phosphorylation but also enhanced MVs production from the cells (Fig. 4A). Pre-treatment of cells with PI3K inhibitor, LY294002 lowered AKT phosphorylation induced by trypsin which was also accompanied by a significant reduction in MVs release (Fig. 4B). In order to correlate both AKT as well as Rab5a activation in the context of MVs generation, we transfected the cells with all three forms (Rab5a WT, DN and CP) of Rab5a followed by LY294002 treatment before the addition of trypsin. Both AKT activation and subsequent MVs generation was analyzed by western blotting (Fig. 4C). The results suggest that LY294002 treatment dramatically lowered trypsin dependent AKT activation and subsequent MVs production in non-transfected as well as Rab5a WT transfected MDA-MB-231 cells. But in case of Rab5a CP transfected cells, LY294002 treatment could not hamper MVs generation although AKT activation significantly reduced. To confirm the chronology of activation further, we have knocked-down Rab5a in MDA-MB-231 cells and checked AKT phosphorylation upon trypsin challenge (Supplementary Fig. 3A,B). The result suggests that Rab5a knock-down had no effect on AKT activation by trypsin. These data imply that Rab5a works somewhere downstream of AKT. Again, Rab5a DN transfected LY294002 treated cells failed to show enhanced reduction in MVs count by trypsin as compared to their lone treatment, which suggests that both AKT and Rab5a are operating in a single signaling cascade. We next analyzed the effect of AKT inhibition in actin polymerization which is absolutely necessary for MVs biogenesis by trypsin (Fig. 4D). G/F-actin ratios suggest that pre-treatment of cells with AKT phosphorylation inhibitor, LY294002 resulted in the reduction of actin polymerization and hence lowered MVs generation.

In sum, the data clearly demonstrate that treatment of MDA-MB-231 cells with trypsin results in immediate activation of AKT in a PI3K dependent manner which is followed by Rab5a activation that critically regulates MVs production by altering cellular actin dynamics.

### Trypsin mediated Rab5a activation promotes migration and invasion of MDA-MB-231 cells

**Figure 5:**
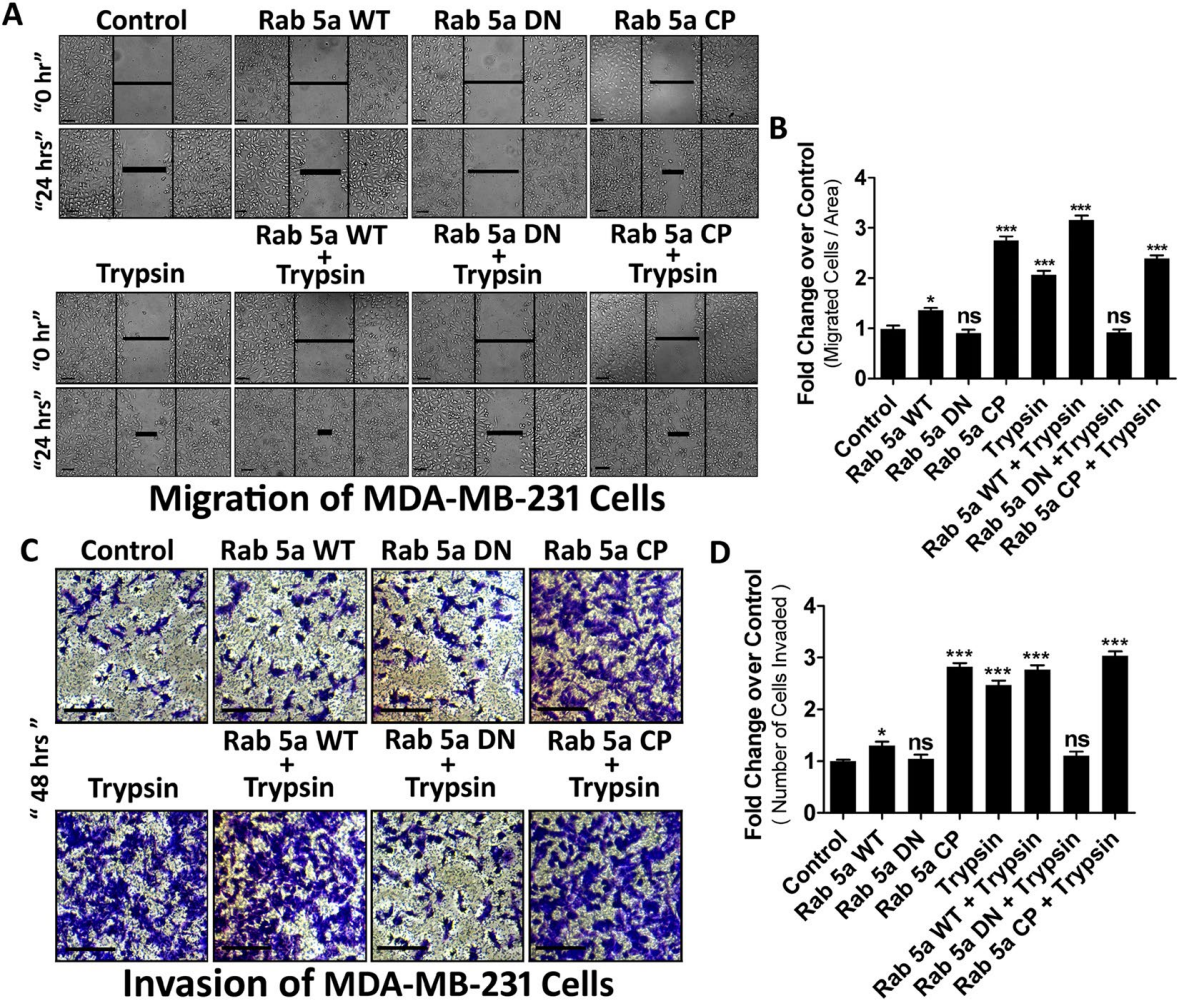
Trypsin Dependent Rab5a Activation Promotes Migration and Invasion of MDA-MB-231 Cells. Cells were transfected with Rab5a WT, Rab5a DN and Rab5a CP and incubated for 48 hours at the end of which serum starvation was given followed by trypsin addition. Migration potential of the cells were analyzed by wound healing assay as described briefly in methods and accordingly quantification was made by GraphPad Prism5 (**A**) and (**B**). Cells were treated with various Rab5a constructs and placed on top of a transwell membrane previously coated with matrigel in serum free media. Cells were treated with trypsin and the lower compartment is filled with complete media. Cells that were invaded through the membrane were fixed and stained with crystal violet solution. Images were taken and number of invaded cells was counted and quantified accordingly (**C**) and (**D**). Data presented over here are as Mean +/− S.E. of the Mean after performing the experiments at least thrice and differences are statistically significant at p < 0.05 using student’s t-test and ‘ns’ represents non-significant differences.

Previous studies have demonstrated that cancer cells produce invadopodia or podosomes like structures in order to invade the extracellular matrix (ECM) and migrate to distant places. Pronounced polymerization of actin microfilament is required to generate such structures^37,38^. We have already shown that Rab5a activation is associated with actin polymerization leading to MVs generation from MDA-MB-231 cells. Hence, we questioned whether the activation of Rab5a has any impact on MDA-MB-231 cell motility. Cells were transfected with various constructs of Rab5a as mentioned before followed by challenge with trypsin. Migration and invasion properties of the cells were estimated by wound healing assay (Fig. 5A,B) and transwell invasion assay (Fig. 5C,D) respectively, as mentioned briefly in methods. Results suggest that the over-expression of Rab5a DN attenuates the trypsin induced cell migration and invasion. Moreover, when the cells were transfected with Rab5a siRNA, trypsin induced cell migration significantly reduced suggesting the active involvement of Rab5a in trypsin mediated MDA-MB-231 cell migration (Supplementary Fig. 4A,B). Both migration and invasion data suggest that Rab5a activation by trypsin not only induces MVs generation from the cell surface but also promotes cancer cell metastasis by virtue of increasing cell migration and invasion.

### Rab5a generated MVs enhance invasion in MCF-7 cells via p38 MAPK activation

**Figure 6:**
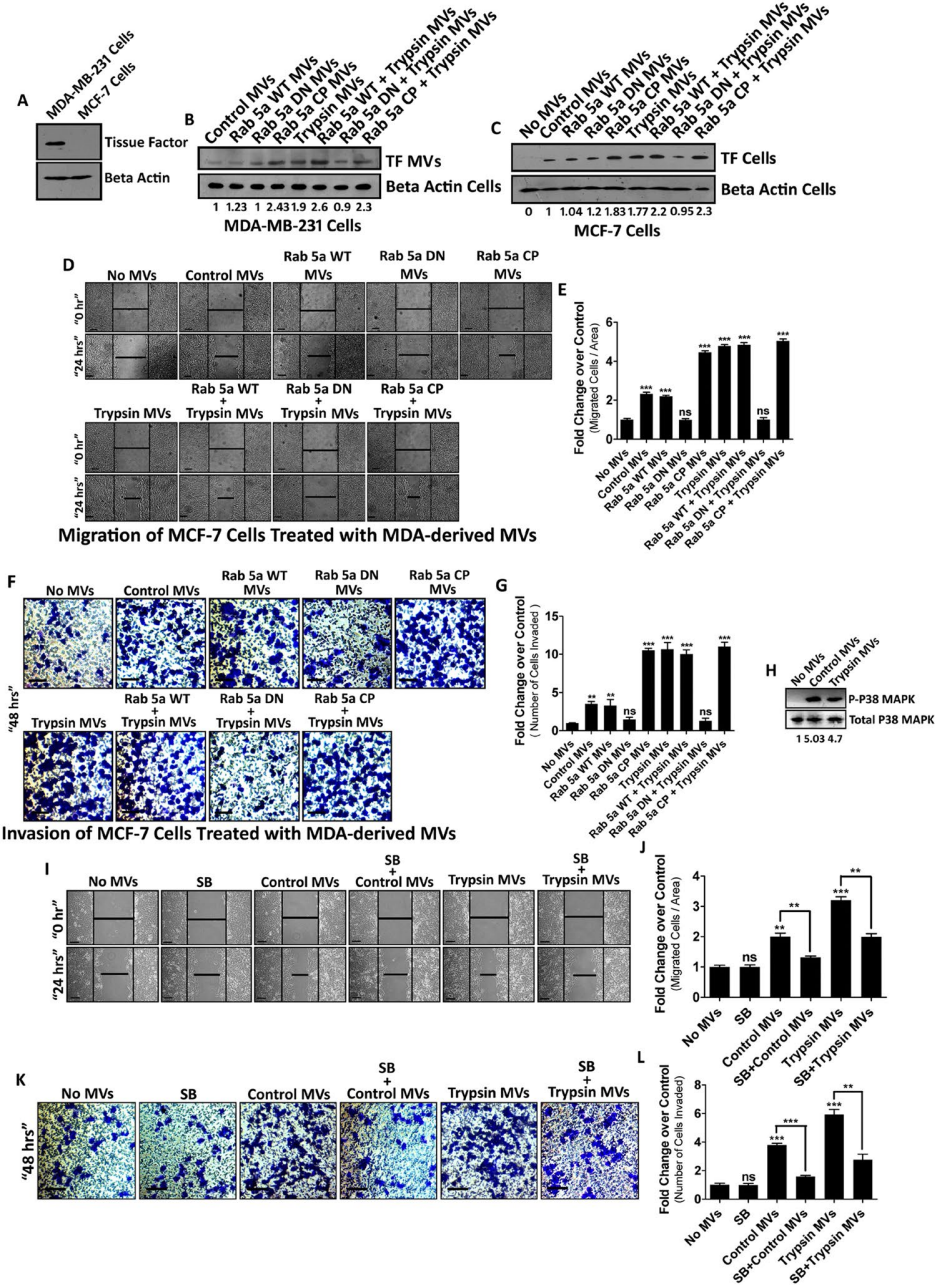
MVs Generated from MDA-MB-231 Cells upon Rab5a Activation Induces MCF-7 Cell Metastasis in A p38 Dependent Manner. Comparative TF level in MDA-MB-231 vs MCF-7 cells (**A**). MVs were isolated from MDA-MB-231 cells after Rab5a transfection and trypsin addition and quantified by western blotting (**B**). MDA-MVs were incubated with MCF-7 cells and MVs fusion was confirmed by analyzing TF in MCF-7 cells (**C**). MVs fused MCF-7 cell migration was assessed by wound healing assay (**D**) and (**E**). Invasive potential of MVs was also verified by transwell invasion assay after fusing the MVs with a non-invasive cell MCF-7 (**F**) and (**G**). MVs were generated from MDA-MB-231 cells after trypsin treatment alongside control and were fused with MCF-7 cells. p38 MAPK phosphorylation was analyzed in these MCF-7 cells by western blotting (**H**). MCF-7 cells were treated previously with p38 MAPK activity inhibitor, SB203580 followed by MDA-derived MVs addition. Migration and invasion of these MCF-7 cells was determined by wound healing assay and transwell invasion assay respectively (**I**–**L**). The experiments were repeated at least for three times. Data presented over here are as Mean +/− S.E. of the Mean and differences are statistically significant at p < 0.05 using student’s t-test and ‘ns’ represents non-significant differences.

Previous studies have documented that MVs contribute to cancer propagation by means of increasing cancer cell motility^17^. To determine the metastatic potential of our MVs generated via Rab5a activation by trypsin from MDA-MB-231 cells, we incubated these MVs with a less metastatic, less aggressive human breast cancer cell line MCF-7. MCF-7 cells bear negligible amount of cell surface TF (Fig. 6A) and the fusion of MVs was confirmed by analyzing the MDA-MVs carrying TF marker (Fig. 6B) in MCF-7 cells (Fig. 6C). Next, we verified both the migration potential as well as invasiveness of the MVs-fused recipient MCF-7 cells by wound healing assay (Fig. 6D,E) and transwell invasion assay (Fig. 6F,G), respectively. The data shows that more relative migration and invasion of MCF-7 cells were taken place when Rab5a activated MDA-cell derived MVs were incorporated into MCF-7. This might be due to the fact that Rab5a activated cells generate more such MVs which upon fusion with MCF-7 cells results in pronounced activation of cell migration and invasion as compared to control.

Previous reports suggest that p38 MAPK plays predominant roles in cell movement^39^. In our study, we have observed that MVs from MDA-MB-231 cells were capable of inducing cell migration and invasion *in vitro*. Naturally, the question arises whether MVs mediated induction of cell movement is dependent on intracellular activation of p38 MAPK or not. First of all, we collected MVs from MDA-MB-231 cells and incubated them with MCF-7. p38 MAPK activation (via phosphorylation) in the recipient MCF-7 cells was verified by western blotting (Fig. 6H). Next, we challenged MCF-7 cells with p38 MAPK activity inhibitor, SB203580 before the addition of MDA-MVs. MCF-7 cell migration and invasion were analyzed by wound healing assay (Fig. 6I,J) and transwell invasion assay (Fig. 6K,L), respectively. The results suggest that MDA-derived MVs induced MCF-7 cell migration and invasion was reversed significantly when recipient MCF-7 cells were treated previously with SB203580. This defines the active involvement of p38 MAPK in MDA-MVs induced MCF-7 cell migration and invasion.

Overall, the results demonstrate that MVs, generated from highly aggressive MDA-MB-231 cells upon trypsin mediated Rab5a activation induces migration and invasion to less aggressive MCF-7 cells in a p38 MAPK dependent manner.

### MVs from breast cancer patients display higher metastatic potential

**Figure 7:**
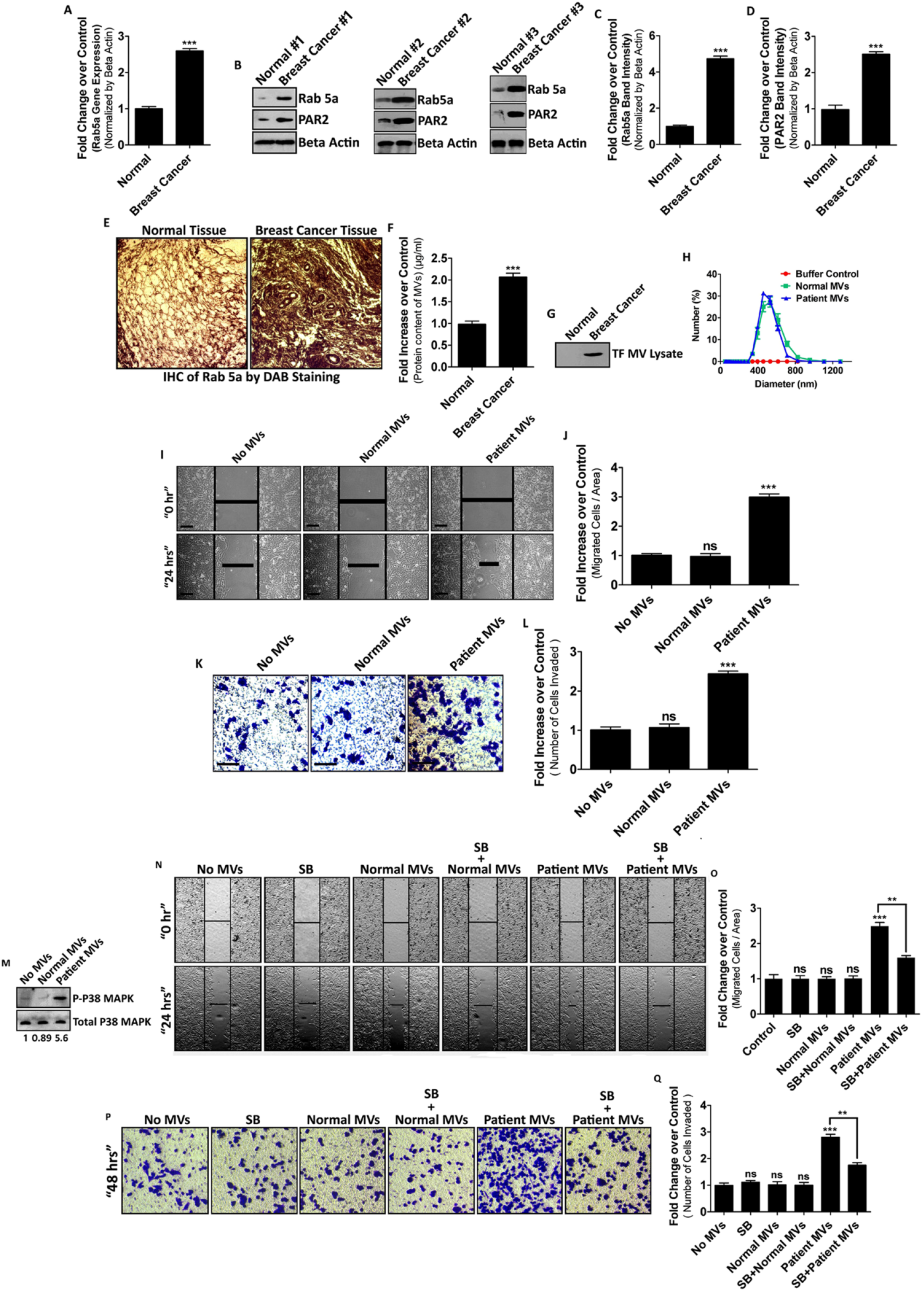
MVs from breast cancer patients display higher metastatic potential. Both normal as well as human breast cancer tissue samples were collected and subjected to analysis of Rab5a expression at transcription level (**A**) and protein level (**B**), (**C**) and (**E**). PAR2 protein expression was also estimated in those tissues (**B**) and (**D**). MVs were isolated from the blood of both breast cancer as well as normal individuals and quantified by Bradford assay and western blotting with TF marker (**F**) and (**G**). DLS study was performed with these MVs to determine their size (**H**). Migration (**I**) and (**J**) as well as invasive (**K**) and (**L**) potential of these blood-borne MVs was analyzed by wound healing assay and transwell invasion assay, respectively. MVs, isolated from the blood samples were fused with MCF-7 cells and p38 MAPK activation was assessed by western blotting (**M**). MCF-7 cells were treated with SB203580 for an hour followed by the addition of blood-derived MVs. Migration and invasion was checked by wound healing assay and transwell invasion assay, respectively to determine the metastatic potential of MVs (**N**–**Q**). The experiments were repeated at least for three times. Data presented over here are as Mean +/− S.E. of the Mean and differences are statistically significant at p < 0.05 using student’s t-test and ‘ns’ represents non-significant differences.

To determine whether our *in vitro* observations have any correlation with real *in vivo* conditions, we have collected both human breast cancer tissue samples and normal breast tissue samples (women aged between 40–45 years) following human ethics committee guidelines. We verified Rab5a gene expression by real time PCR, western blotting and IHC study (both RNA and protein level) in both forms of tissues in a comparative approach (Fig. 7A–C and E). Data showed that Rab5a expression is quite higher in breast cancer tissues as compared to normal tissues which are also accompanied by a relative higher expression of PAR2 in the cancer tissues (Fig. 7B,D). Moreover, breast cancer patients’ blood contains more of TF-bearing MVs than the blood collected from normal healthy individuals as determined by Bradford assay and western blot analyses (Fig. 7F,G). The data also suggest that barely any TF band was visible in the MVs isolated from healthy individuals although MVs protein estimation confirmed the presence of MVs in the samples. To quantify both TF-bearing as well as TF-depleted MVs in the blood samples (both normal and cancer) we employed immunoprecipitation based MVs separation technique as described briefly in methods. The data showed that total MVs population was significantly higher in patient derived blood samples than normal (Supplementary Fig. S5E). TF-bearing MVs percentage was far greater in patient’s blood whereas blood samples of healthy individuals contained more TF-depleted MVs as determined by western blotting and Nile Red counting (Supplementary Fig. 5A-D). These MVs has a diameter range of 400–800 nm (Fig. 7H). We have also studied the metastatic potential of these MVs by wound healing assay (Fig. 7I,J) and transwell invasion assay (Fig. 7K,L). The results suggest that both migration and invasion potential of cancer patient-derived MVs is significantly higher than normal MVs.

Again, we treated MCF-7 cells with both normal and breast cancer patient-derived MVs which showed a significant rise in p38 MAPK phosphorylation in patient-derived MVs incorporated MCF-7 cells unlike normal (Fig. 7M). Migration and invasion analyses with this blood borne MVs suggest that patient-derived MVs were capable of inducing both migration and invasion of recipient MCF-7 cells which was reversed upon introduction of p38 MAPK activity inhibitor, SB203580. This signifies again the contribution of p38 MAPK in MVs induced MCF-7 cell migration and invasion. No such changes in cell behavior were monitored with normal individual-derived MVs incorporated MCF-7 cells (Fig. 7N–Q).

Overall, the data support that PAR2-driven Rab5a mediated release of pro-metastatic MVs generation might also happen in real physiologic conditions and p38 MAPK play a vital role in MVs induced cell migration as well as invasion.

## Discussion

Breast cancer is the second leading cause of cancer death in women worldwide and statistics suggest that one in eight women will develop invasive breast cancer in her life-time at a percentage of approximately 12. The severity of the disease results from the metastasis of tumor from initial tumor forming site to a secondary site which requires the detachment of cancer cells from its origin, degradation of ECM, migration through blood vessels and colonization at a different location. Tumor cells do not perform these alone but assisted by tumor associated cells as well as tumor cell-secreted small nano-vesicles (MVs) which contribute immensely in that process.

**Figure 8:**
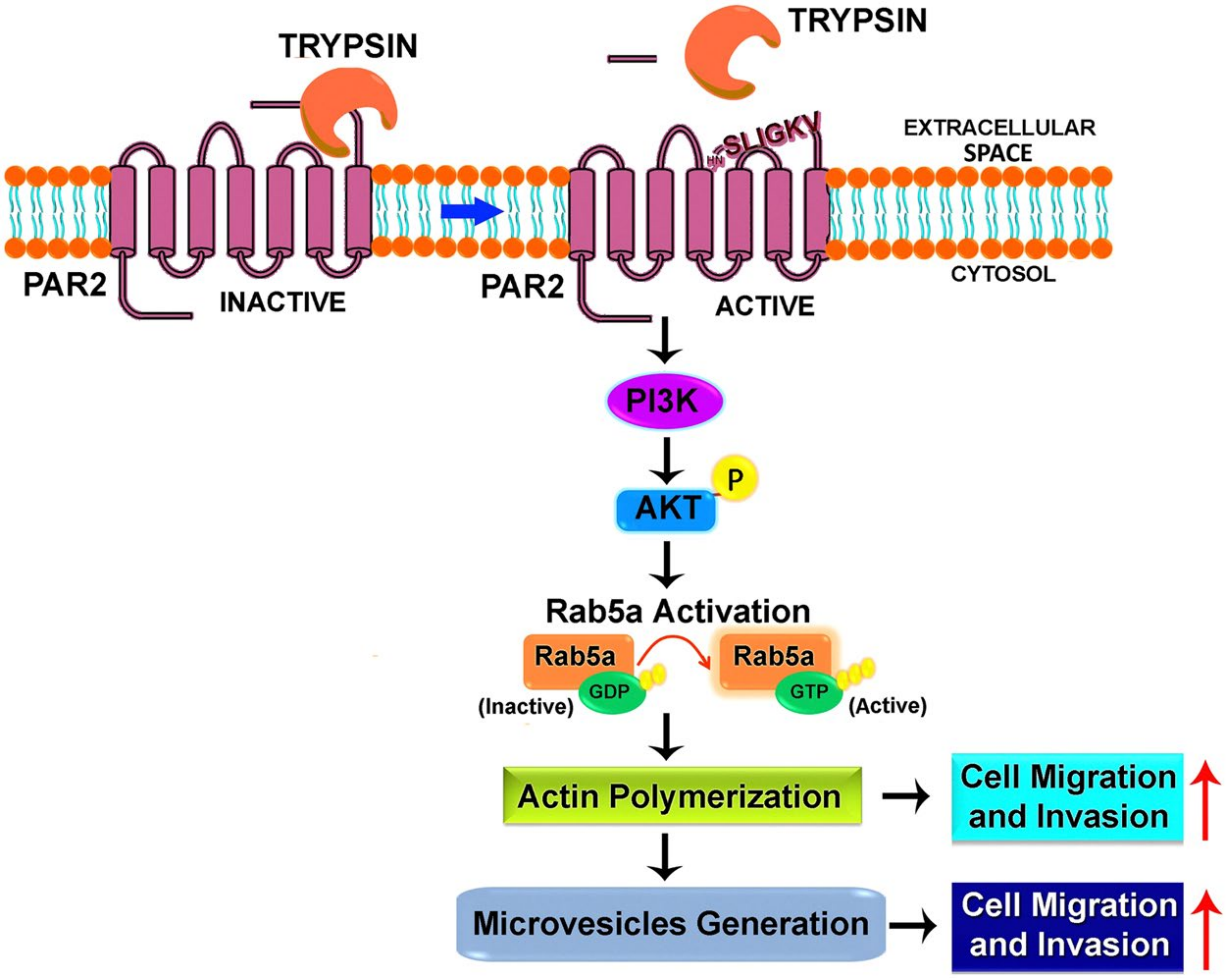
Schematic Diagram Showing That Trypsin Mediated PAR2 Stimulation Promotes Pro-migratory And Pro-invasive MVs Generation via Rab5a Activation. The activation of PAR2 by trypsin triggers PI3K-dependent AKT phosphorylation at Serine 473. This induces the activation of Rab5a from inactive GDP-bound form to active GTP-bound state resulting in actin polymerization which is critical for MVs generation. Rab5a activation also leads to cell migration and invasion. These MVs have the potential to induce cell migration and invasion.

In this study, we demonstrate that trypsin-mediated cleavage of PAR2 leads to the generation of pro-metastatic MVs from human breast cancer cells via the activation of Rab5a. Knock-down of PAR2 significantly decreased trypsin triggered MVs count suggesting that PAR2 activation is essential in MVs biogenesis. Treatment of cells with dominant negative mutant (constitutively inactive; Rab5a DN) of Rab5a also retarded MVs generation by trypsin whereas, constitutively active form of Rab5a (Rab5a CP) enhanced MVs generation significantly irrespective of trypsin addition. Also, knock-down of Rab5a significantly reduced trypsin mediated MVs generation which again confirms that Rab5a actively participates in MVs biogenesis. Interestingly, we have observed that Rab5a activation leads to the polymerization of actin which is critical during cellular bud formation to generate the MVs. Pre-treatment of cells with actin polymerization inhibitor, Latrunculin B significantly reduced trypsin triggered MVs generation suggesting the role of actin cytoskeleton in MVs generation. Knock-down of Rab5a also prevented actin polymerization induced by trypsin demonstrating that trypsin induces Rab5a activation that leads to actin polymerization and subsequent MVs production. This understudied area of PAR2 mediated MVs biogenesis via the activation of Rab5a has been highlighted in the present study. We also demonstrate that treatment of trypsin to MDA-MB-231 cells causes immediate activation of AKT via phosphorylation at Ser 473 residue which ultimately leads to Rab5a activation and thereby influence MVs generation (Fig. 8). Not only Rab5a activation leads to MVs generation, but also it alters cellular actin network in such a way that cell migration as well as invasion is significantly enhanced. When these MVs, collected from MDA-MB-231 cell-supernatant were incorporated into a less migratory, less invasive MCF-7 cells, the migration as well as invasive potential of MCF-7 highly promoted. Non-significant migration and invasion of MCF-7 cells were observed when Rab5a DN transfected MDA-MB-231 cell-secreted MVs were incorporated into MCF-7 cells. A possible reason behind that is less MVs load in Rab5a DN treated cell-supernatant which allows less MVs fusion with recipient MCF-7 cells, thereby showing less migration and invasion.

To determine whether our *in vitro* observations have any correlation with real physiological conditions, we collected blood samples of human breast cancer patients (n = 10) alongside normal healthy women (n = 10) aged between 40–45 years. Analysis of MVs quantification suggests that blood samples collected from cancer patients contained more MVs than the blood from normal healthy individuals. These MVs were also capable of inducing cell migration and invasion as observed in our *in vitro* experiments. Moreover, we noticed that Rab5a and PAR2 are over-expressed in human breast carcinoma tissues as compared to normal breast tissues. This signifies that more pro-metastatic MVs count in cancer patients’ blood may result from PAR2 mediated Rab5a activation as observed intensely in our *in vitro* system. We also demonstrate that the cellular transformations of MCF-7 by means of increase in migration and invasion via MVs are dependent on intracellular activation of p38 MAPK. MVs-dependent both migration and invasion of MCF-7 cells were reduced significantly upon administration of p38 MAPK activity inhibitor, SB203580. These preclinical data provide a scientific evidence for clinical trials that include inhibitors of MVs generation along with existing chemotherapy regimens to restrict breast cancer metastasis.

## Methods

### Cell culture

The human breast cancer cell lines, MDA-MB-231 and MCF-7 were purchased from ATCC and cultured in standard DMEM (HIMEDIA, India). The media was supplemented with 10% FBS (HIMEDIA) and 1% penicillin-streptomycin (Invitrogen). Cells were maintained at 37 °C and 5% CO_2_ level.

### Preparation of plasmid constructs and introduction into cells

Wild-type Rab5a (Rab5a WT) was cloned in pEGFP vector and the dominant-negative (Rab5a DN) (Rab5a: S34N) as well as the constitutively active (Rab5a CP) (Rab5a: Q79L) mutants, were generated by side-directed mutagenesis. Plasmids were transfected into MDA-MB-231 cells by using Lipofectamine 2000 (Invitrogen) as per manufacturer’s guideline and the cells were incubated for 48 hours for sufficient expression of the transfected genes.

### Treatment of cells with trypsin

All the Rab5a transfected/non-transfected cells were grown upto 80% confluency. Serum starvation was given for 2 hours with serum deprived DMEM followed by the addition of trypsin (5 nM; Sigma). For the inhibitor treated experiments, inhibitor like LY294002 (25 µM) or Latrunculin B (200 µM) was introduced to the cells an hour before trypsin addition.

### MVs isolation from cell-supernatant

For MVs isolation, both MDA-MB-231 as well as MCF-7 cells was seeded onto 90 mm dishes. At 40–50% confluency, cells were transfected with various forms of Rab5a gene (WT, DN and CP) alongside control. After 48 hours, trypsin treatment was done followed by overnight incubation at 37 °C. MVs were collected from the culture supernatant by sequential centrifugation procedure as mentioned earlier^34^. Briefly, cell-supernatant was centrifuged at 800 g for 10 minutes (to remove floating cells) followed by 2500 g for 15 minutes (to remove apoptotic bodies and cell debris) and 10,000 g for 30 minutes to isolate the MVs. MVs were washed twice with 1x PBS and proceed for analysis.

### Western blotting

MDA-MB-231 cells were treated with PAR2 activation peptide (SLIGKV-NH_2_; 100 µM; GLBiochem, China) or trypsin followed by an incubation of 15 minutes to determine AKT activation by western blotting with phospho-AKT Ser 473 specific antibodies. Subsequent MVs generation was quantified after 24 hours of ligands addition. Cells were challenged with LY294002 followed by trypsin treatment and AKT phosphorylation was verified by western blotting. AKT phosphorylation was also analyzed of Rab5a plasmids transfected, LY294002 treated MDA-MB-231 cells after trypsin addition. MVs generation was quantified accordingly by using TF antibody against a MVs marker protein, TF. In another approach, MCF-7 cells were incubated for 5 hours with MVs isolated from MDA-MB-231 cells upon trypsin treatment and p38 MAPK activation in MCF-7 was analyzed by western blotting with phospho-p38 MAPK (Thr-180/Tyr-182). Similarly, human blood-derived MVs (both normal and patient) were also incubated with MCF-7 cells to assess cellular p38 MAPK activation.

Protein level analysis was performed by standard western blotting protocol. Briefly, cells or MVs were lysed with 2X laemmli buffer followed by heating at 95 °C for 5 minutes. Proteins were then separated by SDS-PAGE and transferred onto PVDF membrane. Membranes were blocked with 5% non-fat dried-milk in TBS and incubated overnight at 4 °C with primary antibodies against specific proteins (1:1000; Cell Signaling Technology). After washing with TBS-Tween 20 (0.1%), secondary antibody (1:1000; Sigma) incubation was given for an hour followed by washing and development by ECL method.

### Fluorescence microscopic counting

MVs (isolated both from cells as well as human blood) were stained with a lipophilic dye, Nile Red and imaged directly under a fluorescence microscope (Olympus BX61) and from the images MVs number was counted by image J and accordingly graphs were prepared by GraphPad Prism 5.

### Bradford assay

MVs (cell-derived and blood borne) were incubated with lysis buffer [Tris (50 mM), NaCl (150 mM), 1% NP-40, MgCl_2_ (10 mM), DTT (5 mM) and Protease inhibitor (Roche)] for an hour with intermittent agitation and MVs protein was separated by spinning at 16,000 rpm for 15 minutes (to pellet debris). Protein concentration in the supernatant was estimated by standard Bradford assay protocol by measuring O.D. at 595 nm. BSA was used to prepare the standard curve and comparative graphical analysis was performed using GraphPad Prism 5.

### DLS analysis

MVs isolated both from the cells after various treatments as well as from human blood were further analyzed by DLS. MVs were re-suspended in serum free media and upon normalization (by measuring protein content by Bradford assay) were subjected to DLS analysis for determining their size by using Zetasizer Nano ZS (Malvern Particle Size Analyzer). The diameter was measured in dm at 25 °C.

### Small interfering RNA treatment

MDA-MB-231 cells were transfected with 100 nM of control scrambled siRNA or PAR2 siRNA (Sigma) using Lipofectamine 2000. The siRNA sequences are as follows: PAR2 siRNA 1-5′-AGUCGUGAAUCUUGUUCAATT-3′, PAR2 siRNA 2-5′-CUUUGUAUGUCGUGAAGCA-3′, PAR2 scrambled siRNA- 5′-GGACUCUUUAUGGUACGUUUAGAUU-3′. Rab5a siRNA (100 nM) was also introduced to the cells as per the requirements. The sequences are as follows: Rab5a siRNA- 5′-CCAACCAGGAATCAGTGTT-3′, Rab5a scrambled siRNA- 5′-ACTACCGTTGTTATAGGTG-3′. Cells were treated with the siRNAs for 48 hours prior to the experiments for stable knock-down of the target genes which was verified by western blotting. MVs generation was also analyzed in these knocked-down cells by western blotting with TF antibody to determine the effect of these molecules in trypsin-triggered MVs biogenesis.

### Quantifying G-/F-Actin ratio

For G-/F-actin quantification, MDA-MB-231 cells were cultured in 60 mm dishes and over-expressed with Rab5a constructs followed by trypsin addition and 5 hours later, cells were lysed with actin stabilization buffer [PIPES (0.1 M), 30% Glycerol, 5% DMSO, MgSO_4_ (1 mM), EGTA (1 mM), 1% Triton X-100, ATP (1 mM) and protease inhibitor]. Centrifugation was done at 16,000 g for 1^1^/_2_ hours to precipitate the filamentous actin while the supernatant contained the globular form. F-actin was de-polymerized with equal volume of actin destabilization buffer [PIPES (0.1 M), MgSO_4_ (1 mM), CaCl_2_ (10 mM), Cytochalasin D (5 µM)] and both the fractions were subjected to beta actin analysis by western blotting with β-actin antibody [Cell Signaling Technology (13E5); Anti-Rabbit; 1:1000]. Densitometric scanning was done using image J and accordingly graph was prepared by GraphPad Prism5. G-/F-actin ratio was also quantified of trypsin untreated various Rab5a transfected cells. Moreover, cells were challenged with Latrunculin B (Sigma) or LY294002 (Calbiochem) followed by trypsin treatment and G-/F-actin ratio was analyzed to determine the effect of trypsin or AKT in actin polymerization respectively, related to MVs generation. G/F-actin ratio was also quantified in PAR2 as well as Rab5a knocked-down cells after challenging with trypsin to ensure the active participation of these molecules in actin rearrangement.

### Confocal microscopy

MDA-MB-231 cells were over-expressed with all three forms of Rab5a constructs, followed by treatment with trypsin and 5 hours later cells were fixed with 4% para-Formaldehyde. The fixed cells were permeabilized with 0.1% Triton X-100, stained with phalloidin red (Sigma) and visualized under confocal microscope to assess the formation of filamentous actin.

### Wound healing assay

The migration property of cells was analyzed by wound healing assay as described before^40^. Briefly, MDA-MB-231 cells were grown on cover-glasses followed by transfection with various forms of Rab5a and trypsin treatment. A single scratch-line was created on the cell-bed with a small micro-tip. The cells were incubated at 37 °C overnight and after 24 hours, number of cells migrated to the scratched area was counted and accordingly graph was prepared. The assay was also conducted on Rab5a knocked-down cells upon trypsin treatment alongside control to further verify the role of Rab5a in MDA-MB-231 cell migration. MCF7 cell-migration was analyzed by the same approach after treating them with MDA-derived MVs. MCF-7 cells were pre-incubated with p38 MAPK activity blocker, SB203580 (10 µM) for an hour followed by the addition of MDA-derived MVs and migration of the cells was analyzed by wound healing assay. Similarly, MCF-7 cell migration was assessed after fusion with blood-derived MVs and the role of p38 MAPK in this process was also analyzed.

### Transwell invasion assay

Matrigel (Sigma) was paved on the transwell (HIMEDIA, India) membrane (0.8 µm pore size) and after solidification, Rab5a plasmid transfected MDA-MB-231 cells in serum free media were introduced on top of it. Following trypsin treatment, the lower compartment was filled with complete media. After 48 hours, the number of invaded cells in the lower chamber was imaged and quantified after staining with crystal violet solution (0.1% CV, 0.1 M Borate, 2% Ethanol). Invasion of MDA-MVs fused MCF-7 cells was also assessed by the same approach. The graph was prepared accordingly by using GraphPad Prism5. MVs-fused MCF-7 cell invasion was also verified after incorporating SB203580 into the cells to determine the role of p38 MAPK in MDA-MVs-mediated MCF-7 cell invasion. Moreover, MCF-7 cell invasion was also assessed both in presence and absence of SB203580 upon the addition of blood-derived MVs to understand the effect of blood borne MVs in p38 MAPK dependent MCF-7 cell invasion.

### MVs isolation from human blood

Blood samples (10 ml) were collected from ten human epithelial breast cancer patients and ten such normal healthy women aged between 40–45 years obeying human ethics committee rules (Reg. No. ECR/286/INST/WB/2013). Platelets and other cellular parts were discarded after centrifuging the samples at 1000 g for 15 minutes and from the two separate plasma pool (Normal and patient) MVs were isolated by spinning at 10,000 g for 30 minutes (4 °C) as mentioned before^34^. MVs were quantified by both Bradford assay and western blotting with TF antibody and their size was measured by DLS.

### Real-Time PCR analysis

Total RNA was isolated from human breast tissues (normal and cancer) by using TRIZOL reagent (Invitrogen) and reverse transcribed to form first strand cDNA using oligo-dT primer (GCC Biotech). PCR amplification of Rab5a gene was done using Rab5a specific primers: 5′-CAAGAACGATACCATAGCCTAGCAC-3′ (forward), 5′-CTTGCCTCTGAAGTTCTTTAACCC-3′ (reverse). Beta actin was used as loading control, 5′-GGCATGGGTCAGAAGGATTCC-3′ (sense), 5′-AGCACAGCCTGGATAGCAACG-3′ (antisense). cDNA was used in triplicate real time PCR reactions using SYBR Green on a real time PCR machine as per manufacturer’s protocol and accordingly relative Rab5a expression was measured from ΔC_T_. The comparative graphical analysis was performed after repeating the experiment thrice by GraphPad Prism5.

### RNA and protein extraction from human breast tissues

Human breast tissue samples (both normal and cancer; n = 10 each) were collected from Netaji Subhash Chandra Bose Cancer Research Institute, Kolkata. Equal weight of both the tissues (500 mg) was taken, pulverized using tissue homogenizer and total RNA was extracted by conventional TRIZOL method. For protein extraction, the tissues (200 mg) were homogenized in lysis buffer with freshly added protease inhibitors (DTT, leupeptin and aprotinin) followed by centrifugation at 25,000 g for 20 minutes. Protein concentrations in the supernatant were measured by Bradford assay and proceed further for analysis.

### Immunohistochemistry

Immunohistochemistry was performed as described earlier^28^. Briefly, paraffinized tissue-sections were passed through various ethanol concentrations and wash buffer (0.05 M TBS, 0.01% Tween 20) followed by incubation with Target Retrieval Solution (10 mM sodium citrate, 0.05% Tween 20; pH 6) at 95 °C for 15 minutes. After blocking, the tissues were incubated with anti-Rab5a antibody (1:250) for 3 hours followed by treatment with HRP-linked secondary antibody. The sections were treated with DAB solution (with H_2_O_2_) and imaged directly by a light microscope (INVI, MAGNUS).

### Separation of TF-positive and TF-negative MVs by immunoprecipitation

To separate TF-containing MVs from the entire MVs population of human blood (normal and cancer), protein A/G agarose beads (Santa cruz) were used. First of all, MVs (isolated from the blood of normal and breast cancer patients separately; n = 10 each), re-suspended in serum free DMEM (1 ml) were incubated for 2 hours with TF-antibody (20 µg) with continuous agitation. Then the beads (20 µl) were also incubated with this for another 2 hours. The mixture was then centrifuged at 1000 g for 5 minutes to pellet down beads with MVs. Both the supernatants (normal and cancer) were centrifuged at 10,000 g for 30 minutes to collect TF-depleted MVs. Immunoprecipitated pellets (normal and cancer) were treated with low pH Glycine (pH~2–3) to separate MVs from antibody bound beads. The beads were then separated by spinning at 1000 g for 5 minutes and the supernatants (suppose to contain TF-bearing MVs) were centrifuged again at 10,000 g for 30 minutes to isolate TF-MVs. All four MVs populations (+/−TF; normal/cancer) were analyzed further by Nile Red staining and western blotting with TF-specific antibody.

### Statement of ethical approval or consent

Blood samples and human breast tissue samples (both normal and cancer) were obtained from Netaji Subhash Chandra Bose Cancer Research Institute, Kolkata. All methods were performed in accordance with relevant guidelines and regulations. The experimental protocols were approved by the committee of Netaji Subhash Chandra Bose Cancer Research Institute, Kolkata (Reg. No. ECR/286/INST/WB/2013). The informed consent was obtained from all participants and/or their legal guardians.

### Statistical analysis

All the techniques applied here are representative of at least three (n ≥ 3) independent experiments. The data presented here are as mean ±S.E of the mean and the differences are considered to be statistically significant at p < 0.05 using student’s t-test. ‘ns’ indicates non-significant differences. Statistical analyses were performed by using GraphPad Prism5.

## Electronic supplementary material

Supplementary Information

## References

[1] Hargett LA, Bauer NN (2013). On the origin of microparticles: From &quot;platelet dust&quot; to mediators of intercellular communication. Pulm. Circ..

[2] Raposo G, Stoorvogel W (2013). Extracellular vesicles: exosomes, microvesicles, and friends. J. Cell Biol..

[3] Singh A (2016). Exosome-mediated Transfer of αvβ3 Integrin from Tumorigenic to Nontumorigenic Cells Promotes a Migratory Phenotype. Mol. Cancer Res..

[4] Al-Nedawi K (2008). Intercellular transfer of the oncogenic receptor EGFRvIII by microvesicles derived from tumour cells. Nat. Cell Biol..

[5] Li J (2013). Microvesicle-mediated transfer of microRNA-150 from monocytes to endothelial cells promotes angiogenesis. J. Biol. Chem..

[6] Valadi H (2007). Exosome-mediated transfer of mRNAs and microRNAs is a novel mechanism of genetic exchange between cells. Nat. Cell Biol..

[7] Salido-Guadarrama I, Romero-Cordoba S, Peralta-Zaragoza O, Hidalgo-Miranda A, Rodríguez-Dorantes M (2014). MicroRNAs transported by exosomes in body fluids as mediators of intercellular communication in cancer. Onco. Targets. Ther..

[8] Yáñez-Mó M (2015). Biological properties of extracellular vesicles and their physiological functions. J. Extracell. vesicles.

[9] Hulsmans M, Holvoet P (2013). MicroRNA-containing microvesicles regulating inflammation in association with atherosclerotic disease. Cardiovasc. Res..

[10] Blum A (2009). The possible role of red blood cell microvesicles in atherosclerosis. Eur. J. Intern. Med..

[11] Lemoinne S (2014). The emerging roles of microvesicles in liver diseases. Nat. Rev. Gastroenterol. Hepatol..

[12] Sluijter JPG, Verhage V, Deddens JC, van den Akker F, Doevendans PA (2014). Microvesicles and exosomes for intracardiac communication. Cardiovasc. Res..

[13] Favaro E (2014). Human mesenchymal stem cell-derived microvesicles modulate T cell response to islet antigen glutamic acid decarboxylase in patients with type 1 diabetes. Diabetologia.

[14] Mocharla P (2013). AngiomiR-126 expression and secretion from circulating CD34(+) and CD14(+) PBMCs: role for proangiogenic effects and alterations in type 2 diabetics. Blood.

[15] Antonyak MA (2011). Cancer cell-derived microvesicles induce transformation by transferring tissue transglutaminase and fibronectin to recipient cells. Proc. Natl. Acad. Sci. USA.

[16] Gatti S (2011). Microvesicles derived from human adult mesenchymal stem cells protect against ischaemia-reperfusion-induced acute and chronic kidney injury. Nephrol. Dial. Transplant..

[17] Wang T (2014). Hypoxia-inducible factors and RAB22A mediate formation of microvesicles that stimulate breast cancer invasion and metastasis. Proc. Natl. Acad. Sci. USA.

[18] Jordens I, Marsman M, Kuijl C, Neefjes J (2005). Rab Proteins, Connecting Transport and Vesicle Fusion. Traffic.

[19] Gulappa T, Clouser CL, Menon KMJ (2011). The role of Rab5a GTPase in endocytosis and post-endocytic trafficking of the hCG-human luteinizing hormone receptor complex. Cell. Mol. Life Sci..

[20] Yang P-S (2011). Rab5A is associated with axillary lymph node metastasis in breast cancer patients. Cancer Sci..

[21] Roy A (2017). Coagulation factor VIIa-mediated protease-activated receptor 2 activation leads to β-catenin accumulation via the AKT/GSK3β pathway and contributes to breast cancer progression. J. Biol. Chem. jbc.M.

[22] Milia AF (2002). Protease-activated receptor-2 stimulates angiogenesis and accelerates hemodynamic recovery in a mouse model of hindlimb ischemia. Circ. Res..

[23] Hu L (2013). TF/FVIIa/PAR2 promotes cell proliferation and migration via PKCα and ERK-dependent c-Jun/AP-1 pathway in colon cancer cell line SW620. Tumour Biol..

[24] Tanaka Y, Sekiguchi F, Hong H, Kawabata A (2008). PAR2 triggers IL-8 release via MEK/ERK and PI3-kinase/Akt pathways in GI epithelial cells. Biochem. Biophys. Res. Commun..

[25] Maganto-Garcia E, Punzon C, Terhorst C, Fresno M (2008). Rab5 activation by Toll-like receptor 2 is required for Trypanosoma cruzi internalization and replication in macrophages. Traffic.

[26] Green TM, Alpaugh ML, Barsky SH, Rappa G, Lorico A (2015). Breast Cancer-Derived Extracellular Vesicles: Characterization and Contribution to the Metastatic Phenotype. Biomed Res. Int..

[27] Yang L (2015). Proteinase-activated receptor 2 promotes cancer cell migration through RNA methylation-mediated repression of miR-125b. J. Biol. Chem..

[28] Su S (2009). Proteinase-activated receptor 2 expression in breast cancer and its role in breast cancer cell migration. Oncogene.

[29] Liu S, Chen X, Zheng H, Shi S, Li Y (2011). Knockdown of Rab5a expression decreases cancer cell motility and invasion through integrin-mediated signaling pathway. J. Biomed. Sci..

[30] Sevigny LM (2011). Interdicting protease-activated receptor-2-driven inflammation with cell-penetrating pepducins. Proc. Natl. Acad. Sci. USA.

[31] Al-Ani B, Hollenberg MD (2003). Selective tryptic cleavage at the tethered ligand site of the amino terminal domain of proteinase-activated receptor-2 in intact cells. J. Pharmacol. Exp. Ther..

[32] Frittoli E (2014). A RAB5/RAB4 recycling circuitry induces a proteolytic invasive program and promotes tumor dissemination. J. Cell Biol..

[33] Albiges-Rizo C, Destaing O, Fourcade B, Planus E, Block MR (2009). Actin machinery and mechanosensitivity in invadopodia, podosomes and focal adhesions. J. Cell Sci..

[34] Muralidharan-Chari V (2009). ARF6-regulated shedding of tumor cell-derived plasma membrane microvesicles. Curr. Biol..

[35] Dutra-Oliveira A, Monteiro RQ, Mariano-Oliveira A (2012). Protease-activated receptor-2 (PAR2) mediates VEGF production through the ERK1/2 pathway in human glioblastoma cell lines. Biochem. Biophys. Res. Commun..

[36] Iablokov V (2014). Proteinase-activated receptor 2 (PAR2) decreases apoptosis in colonic epithelial cells. J. Biol. Chem..

[37] Bearer EL (1993). Role of Actin Polymerization in Cell Locomotion: Molecules and Models. Am. J. Respir. Cell Mol. Biol..

[38] Yamaguchi H, Condeelis J (2007). Regulation of the actin cytoskeleton in cancer cell migration and invasion. Biochim. Biophys. Acta - Mol. Cell Res..

[39] Hedges JC (1999). A role forp38(MAPK)/HSP27 pathway in smooth muscle cell migration. J. Biol. Chem..

[40] Rodriguez LG, Wu X, Guan J-L (2005). Wound-healing assay. Methods Mol. Biol..

