# Supplementary material for: The Protease Activated Receptor2 Promotes Rab5a Mediated Generation of Pro-metastatic Microvesicles
Source: Sci Rep. 2018 May 9;8:7357. doi: 10.1038/s41598-018-25725-w (PMC5943449; doi:10.1038/s41598-018-25725-w)
Supplement: Supplementary file 1 — Supplementary Information [file 41598_2018_25725_MOESM1_ESM.docx]

**The Protease Activated Receptor2 Promotes Rab5a Mediated Generation of Pro-metastatic Microvesicles**

**Kaushik Das^1^, Ramesh Prasad^1^, Sreetama Roy^1^, Ashis Mukherjee^2^ and Prosenjit Sen^1,*^**

**^1^**Department of Biological Chemistry, Indian Association for the Cultivation of Science, Kolkata-700032, India

**^2^**Netaji Subhash Chandra Bose Cancer Research Institute, Kolkata-700016, India

***Correspondence**: Prosenjit Sen, Department of Biological Chemistry, Indian Association for the Cultivation of Science, 2A & 2B Raja S. C. Mullick Road, Jadavpur, Kolkata-700032, India,

Fax: +913324732805

Tel.: +913324734971 (Ext. 1518) / 8981244884

E-mail: bcps@iacs.res.in

**Supplementary Figures**

**Supplementary Figure S1**

**
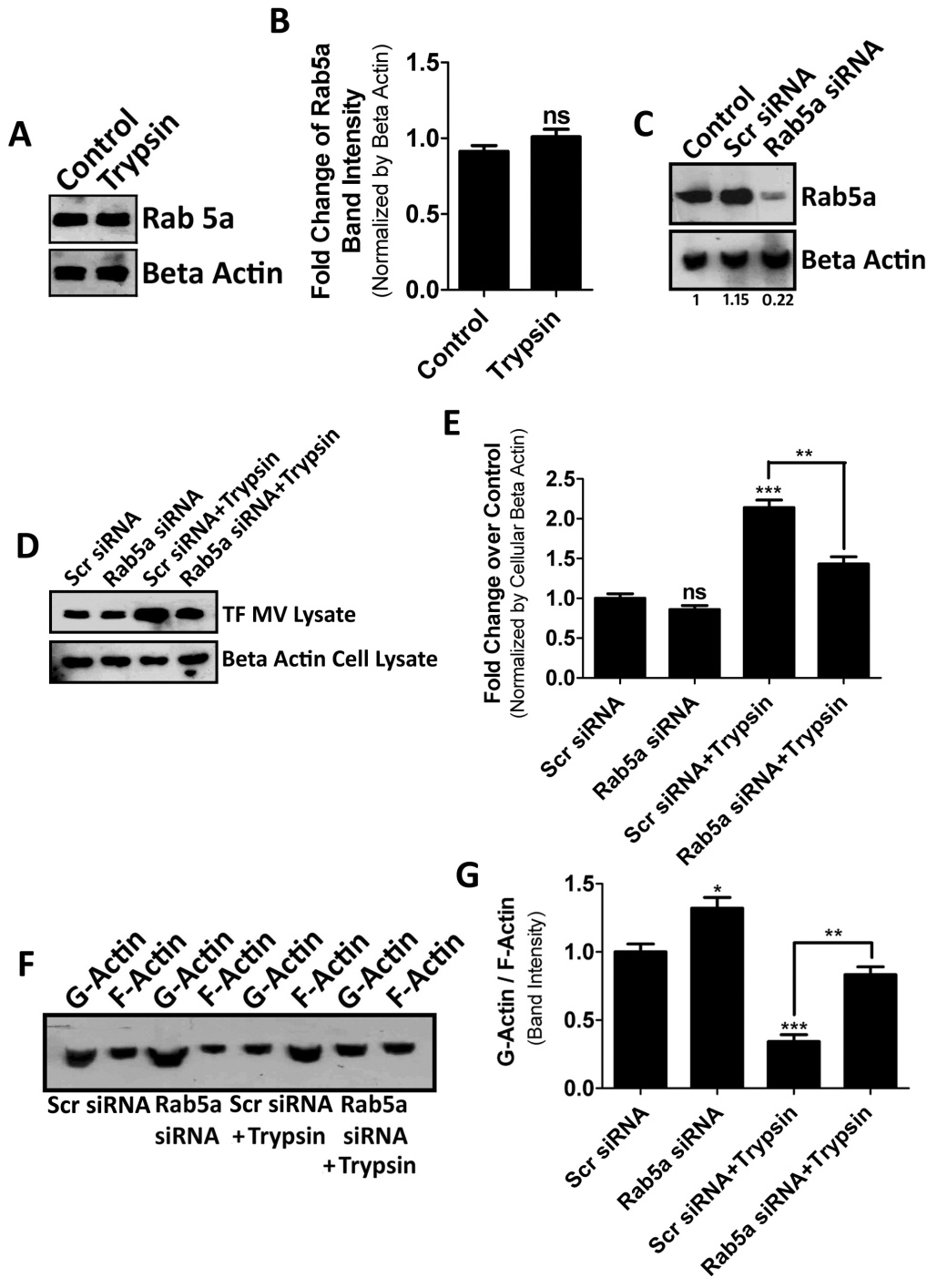
**

**Supplementary Figure S1.** Knock-down of Rab5a Impairs Trypsin-mediated Microvesicles (MVs) Generation from MDA-MB-231 Cells and Also Affecting Actin Polymerization. (**A**). Cells were treated with 5 nM of trypsin alongside an untreated control and after 24 hours of incubation endogenous Rab5a expression was analyzed by western blotting and (**B**). quantification was made by GraphPad Prism5 after measuring band intensity by ImageJ. (**C**). Cells were transfected with 100 nM of Rab5a siRNA alongside scrambled control with the help of Lipofectamine 2000. 48 hours later, Rab5a knock-down was analyzed at protein level by western blotting. (**D**). Both control cells and Rab5a knocked-down cells were challenged with trypsin and after 24 hours MVs were isolated from the supernatant and quantified by western blotting with MVs marker TF. (**E**). Band intensity was measured by Image J and accordingly quantitative micrograph was prepared by GraphPad Prism5. (**F**). Both control as well as Rab5a knocked-down cells were challenged with trypsin and after 5 hours of treatment, cellular G/F actin was assessed by western blotting and (**G**). accordingly quantification was made to prepare the graph. Data presented over here are as Mean +/- S.E. of the Mean and differences are statistically significant at p <0.05 using students’t-test after repeating the experiments at least thrice.

**Supplementary Figure S2**

**
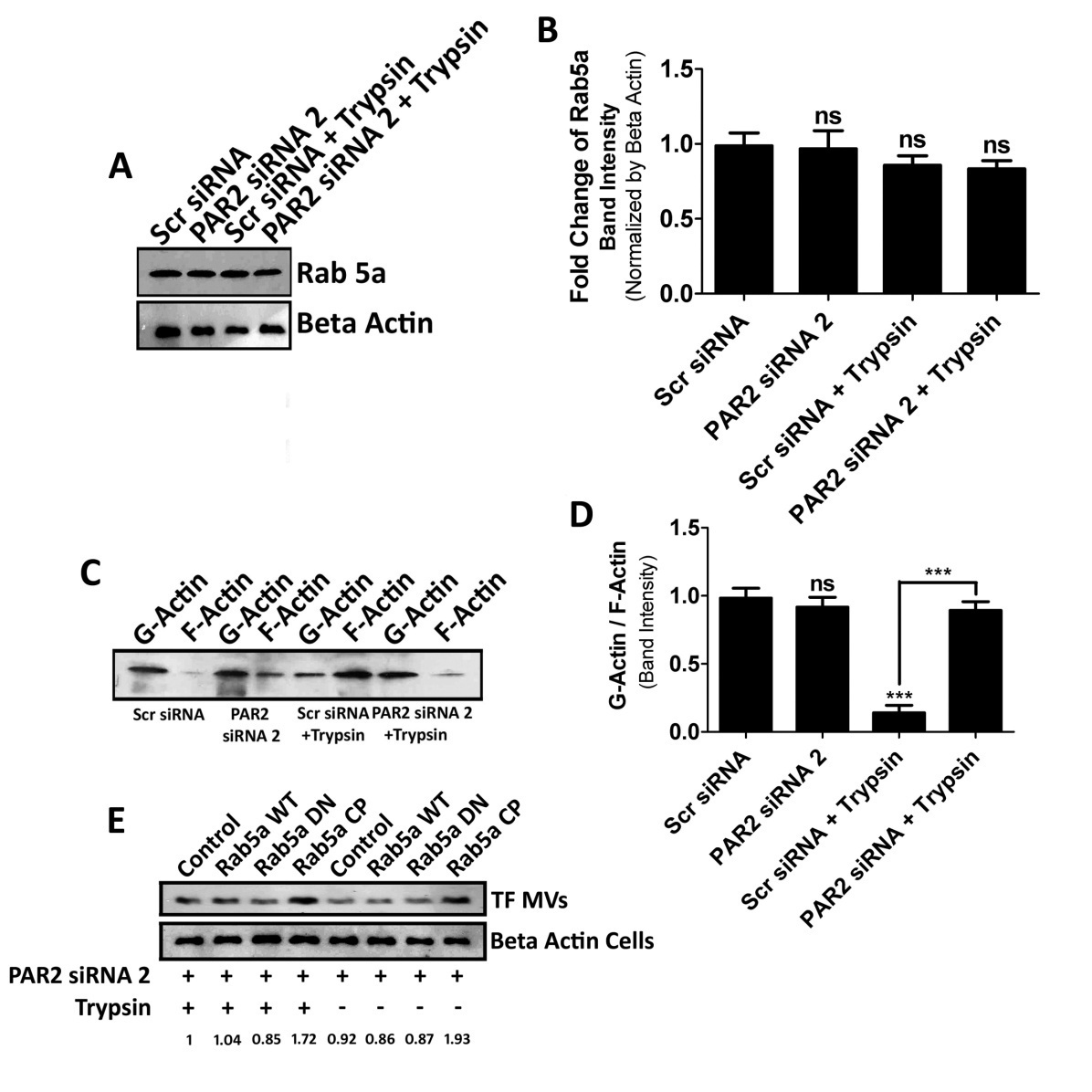
**

**Supplementary Figure S2.** PAR2 Knock-down Alters Trypsin-induced Actin Polymerization Without Affecting Endogenous Rab5a Expression and the Effect of Rab5a Activation in Trypsin-mediated MVs Generation in PAR2 Knocked-down Cells. (**A**). MDA-MB-231 cells were transfected with PAR2 siRNA 2 (100 nM) along with a scrambled control and after 48 hours endogenous Rab5a expression was analyzed at protein level by western blotting. (**B**). Quantitative estimation was carried out by Image J to prepare micrograph by GraphPad Prism5. (**C**) and (**D**). PAR2 knocked-down cells were challenged with trypsin and after 5 hours G/F ratio was quantified to assess the level of actin polymerization. (**E**). Cells were transfected with PAR2 siRNA 2 followed by over-expression of Rab5a constructs (WT, DN and CP). MVs were isolated from the cell supernatant after treatment with trypsin alongside untreated control and quantified by western blotting with MVs marker, TF. Data presented over here are as Mean +/- S.E. of the Mean and differences are statistically significant at p <0.05 using students’t-test after repeating the experiments for at least three times.

**Supplementary Figure S3**


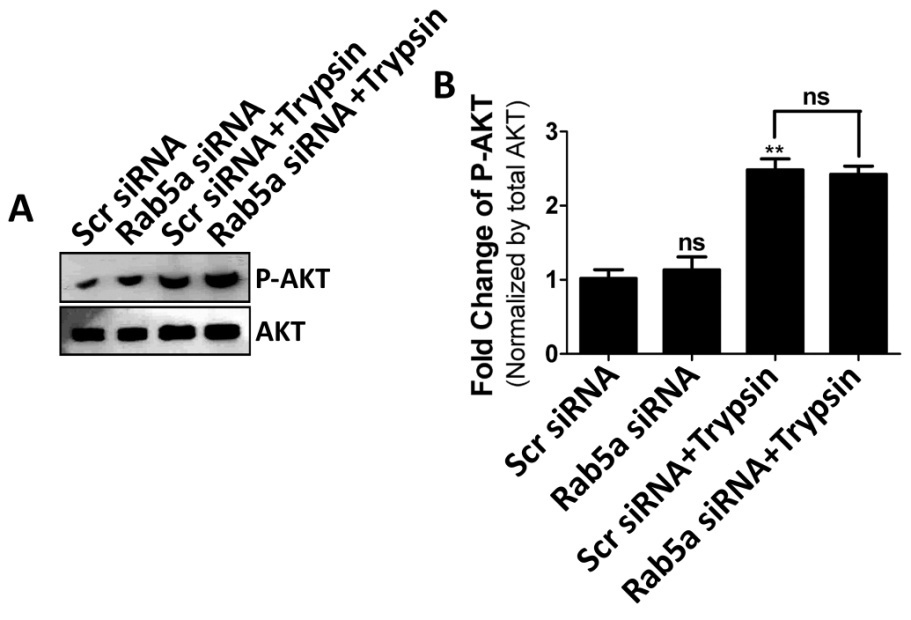


**Supplementary Figure S3.** The Effect of Rab5a Knock-down on Trypsin-mediated AKT Activation in MDA-MB-231 cells. (**A**). Cells were transfected with Rab5a siRNA alongside scrambled control. Both control as well as Rab5a knocked-down cells was challenged with trypsin for 10 mins and AKT phosphorylation was analyzed by western blotting. (**B**). Band intensity was measured by Image J and accordingly micrograph is generated by GraphPad Prism5. Data presented over here are as Mean +/- S.E. of the Mean and differences are statistically significant at p <0.05 using students’t-test after performing the experiments at least thrice.

**Supplementary Figure S4**

**
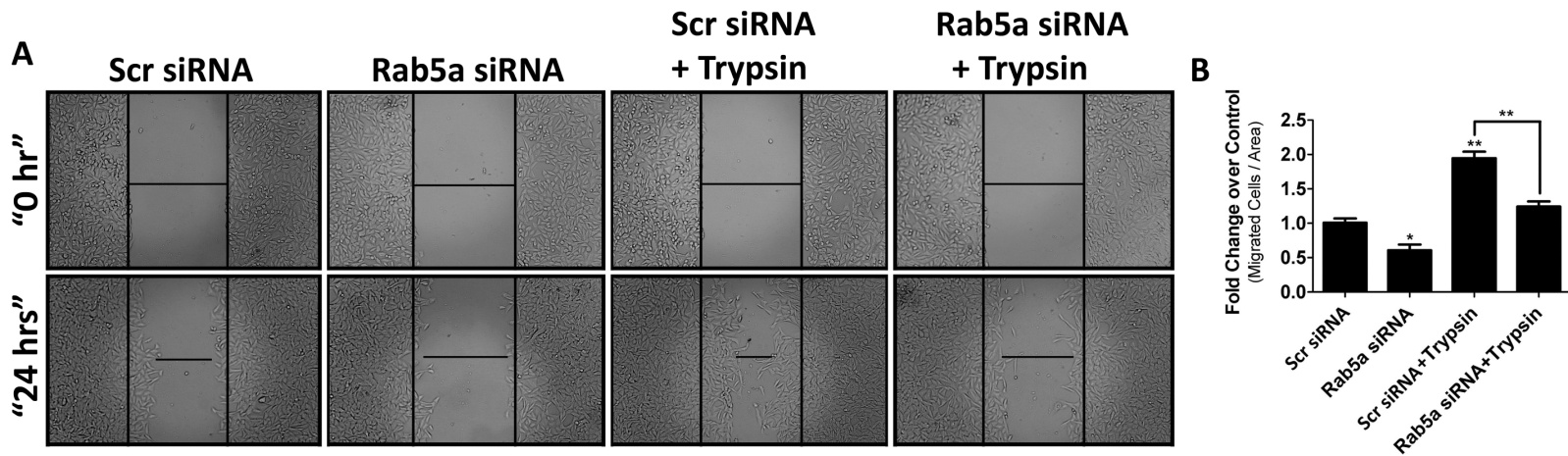
**

**Supplementary Figure S4.** Knock-down of Rab5a Impedes Trypsin-induced Migration of MDA-MB-231 cells. (**A**). Cells were transfected with Rab5a siRNA alongside a scrambled control and after 48 hours of incubation, migration potential of the cells was analyzed upon trypsin challenge by wound healing assay. (**B**). Cells migrated to the scratched area were quantified and accordingly graph was prepared by GraphPad Prism5. Data presented over here are as Mean +/- S.E. of the Mean and differences are considered to be statistically significant at p <0.05 using students’t-test upon repeating the experiments thrice.

**Supplementary Figure S5**

**
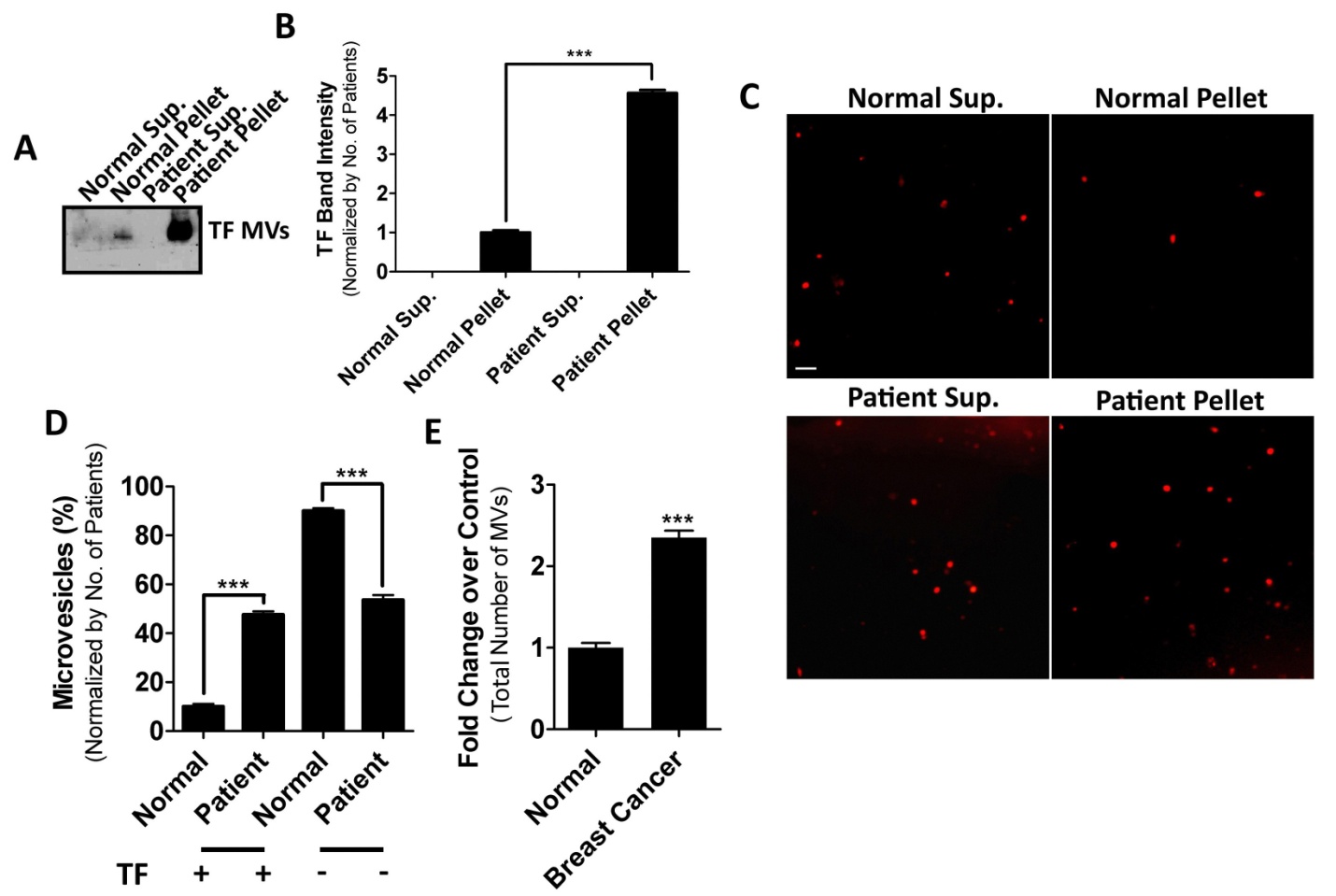
**

**Supplementary Figure S5.** Analysis of Both TF-containing as well as TF-depleting MVs Isolated from the Blood of Human Breast Carcinoma Patients and Normal Healthy Individuals in a Comparative Manner. Blood samples were collected from both human breast carcinoma patients and normal healthy individuals from which MVs were isolated separately. Both MVs populations were incubated with TF-antibody for 2 hours after which protein A/G agarose beads were incubated with it for another 2 hours with continuous agitation. TF-MVs attached beads were separated by centrifugation at 1000g for 5 minutes. The supernatant (both normal and patient) was subjected to MVs isolation (TF-depleted) whereas among the pellet (both normal and patient) antibody-tagged beads were removed from the MVs (TF-positive) by low pH Glycine (pH~2-3) solution. The beads were separated by spinning again at 1000g for 5 minutes whereas from the supernatant MVs were isolated as described in briefly in Methods. All four MVs populations were subjected to quantification by (**A**) and (**B**). western blotting with TF-antibody and (**C**) and (**D**). Nile Red staining method. Total MVs count was also measured by Nile Red staining (**E**). Data presented over here are as Mean +/- S.E. of the Mean and differences are statistically significant at p <0.05 using students’t-test after repeating the experiments at least three times.

**Supplementary Figure S6**


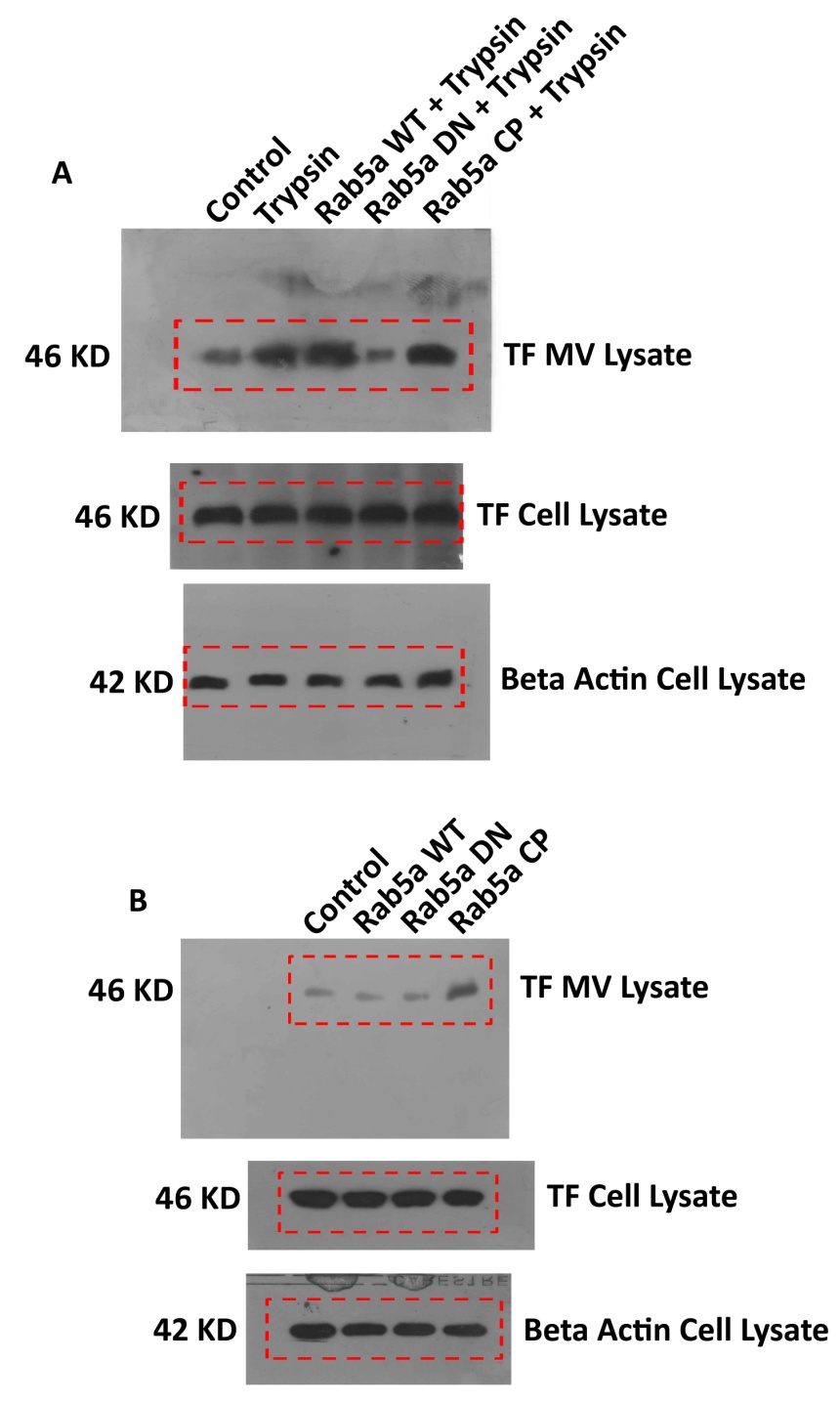


**Supplementary Figure S6.** Full length blots of Figure 1B (Represented here as A) and Figure 1D (Represented here as B). The red dotted lines indicate the cropping locations.

**Supplementary Figure S7**


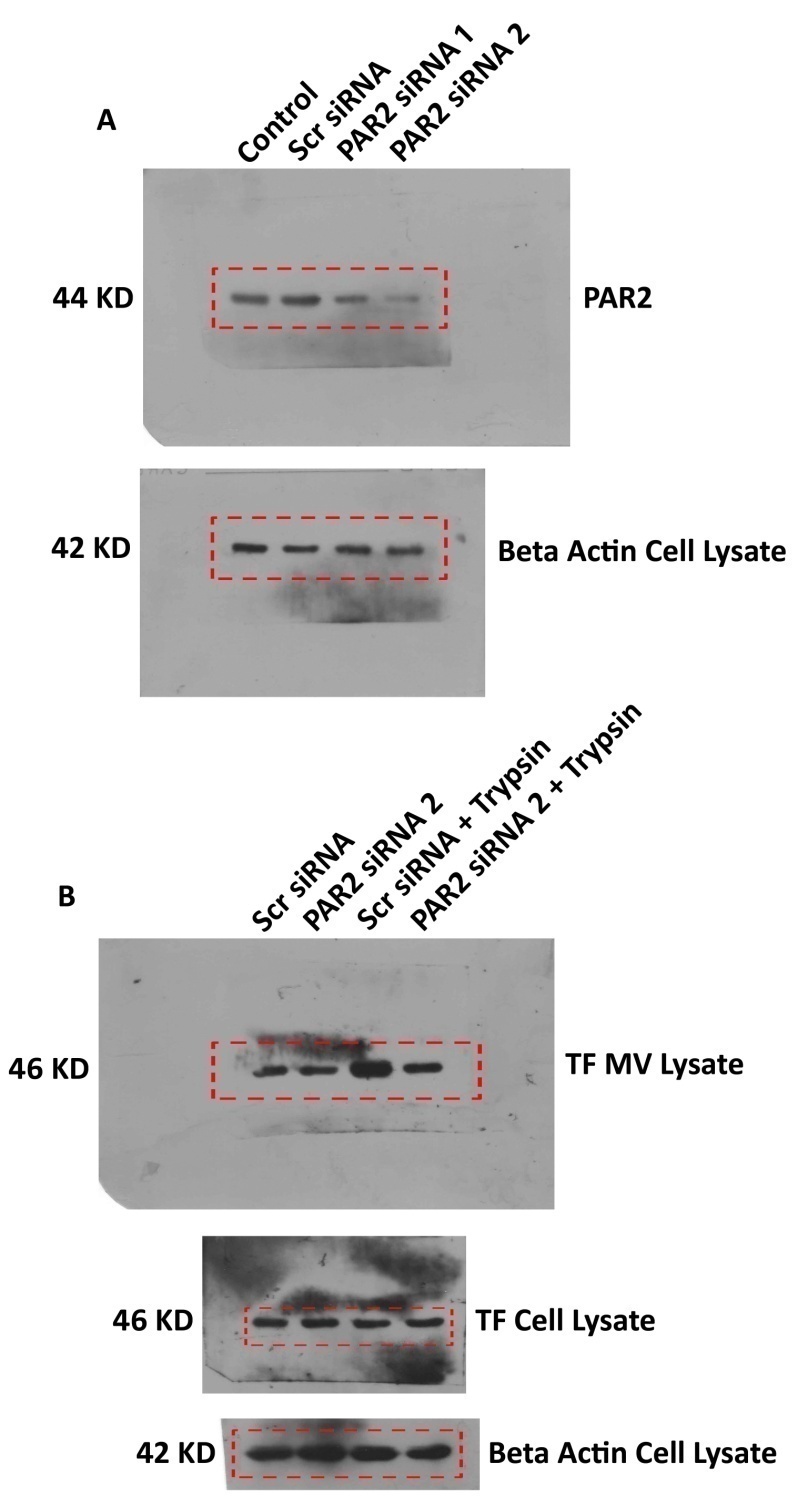


**Supplementary Figure S7.** Full length blots of Figure 2A (Represented here as A) and Figure 2C (Represented here as B). The red dotted lines indicate the cropping locations.

**Supplementary Figure S8**


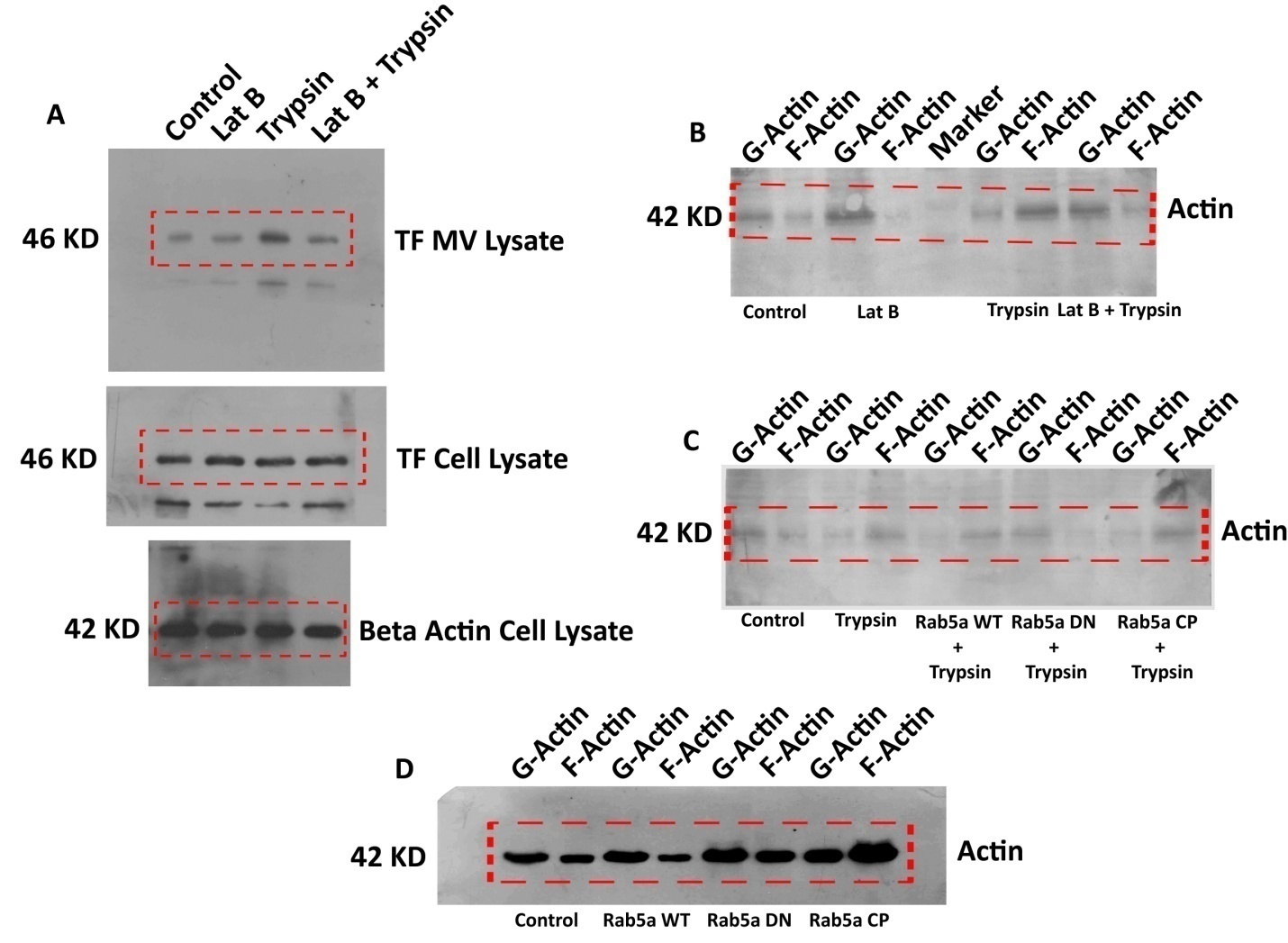


**Supplementary Figure S8.** Full length blots of Figure 3A (Represented here as A), Figure 3C (Represented here as B), Figure 3E (Represented here as C) and Figure 3G (Represented here as D). The red dotted lines indicate the cropping locations.

**Supplementary Figure 9**


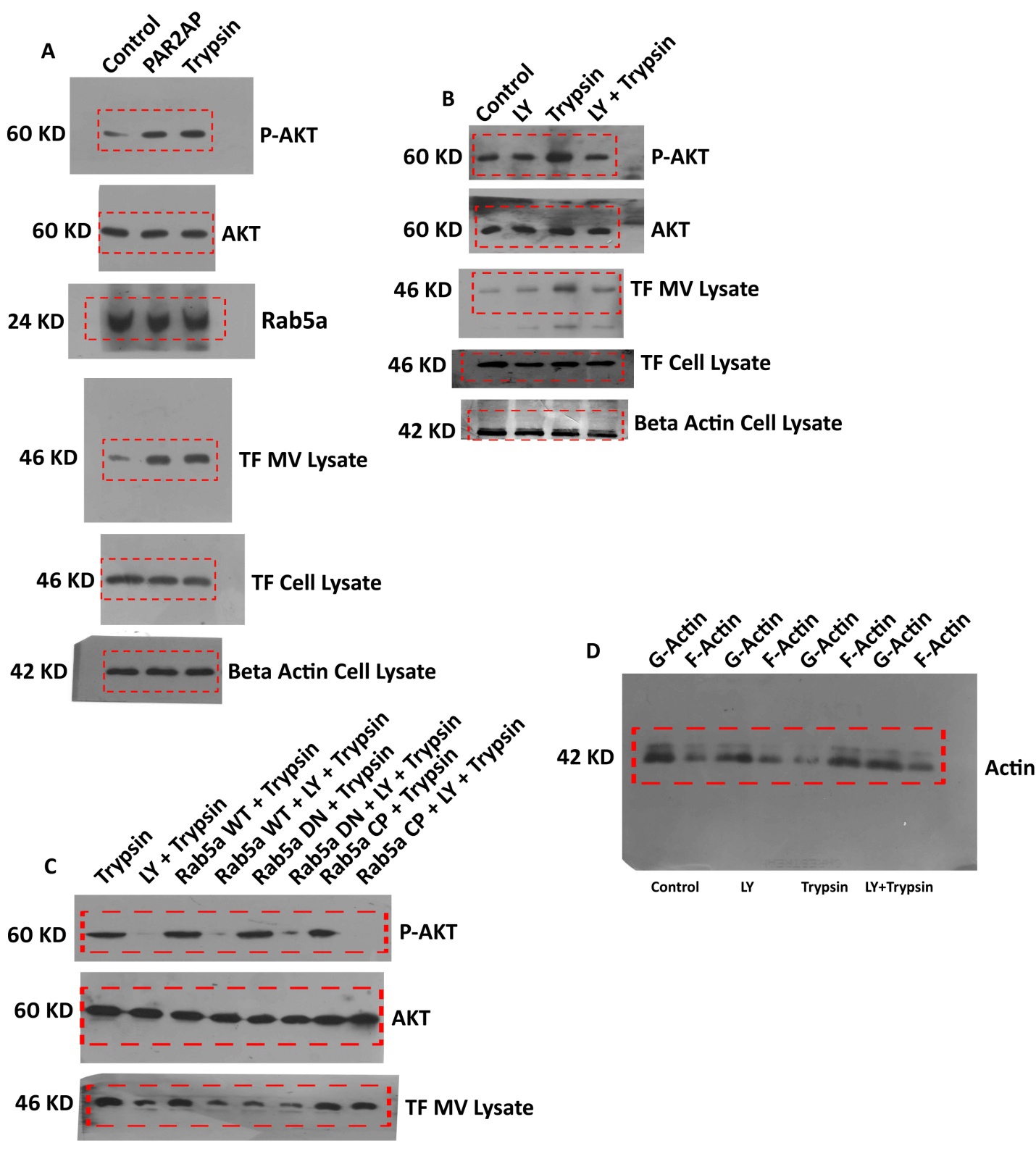


**Supplementary Figure S9.** Full length blots of Figure 4A (Represented here as A), Figure 4B (Represented here as B), Figure 4C (Represented here as C) and Figure 4D (Represented here as D). The red dotted lines indicate the cropping locations.

**Supplementary Figure S10**


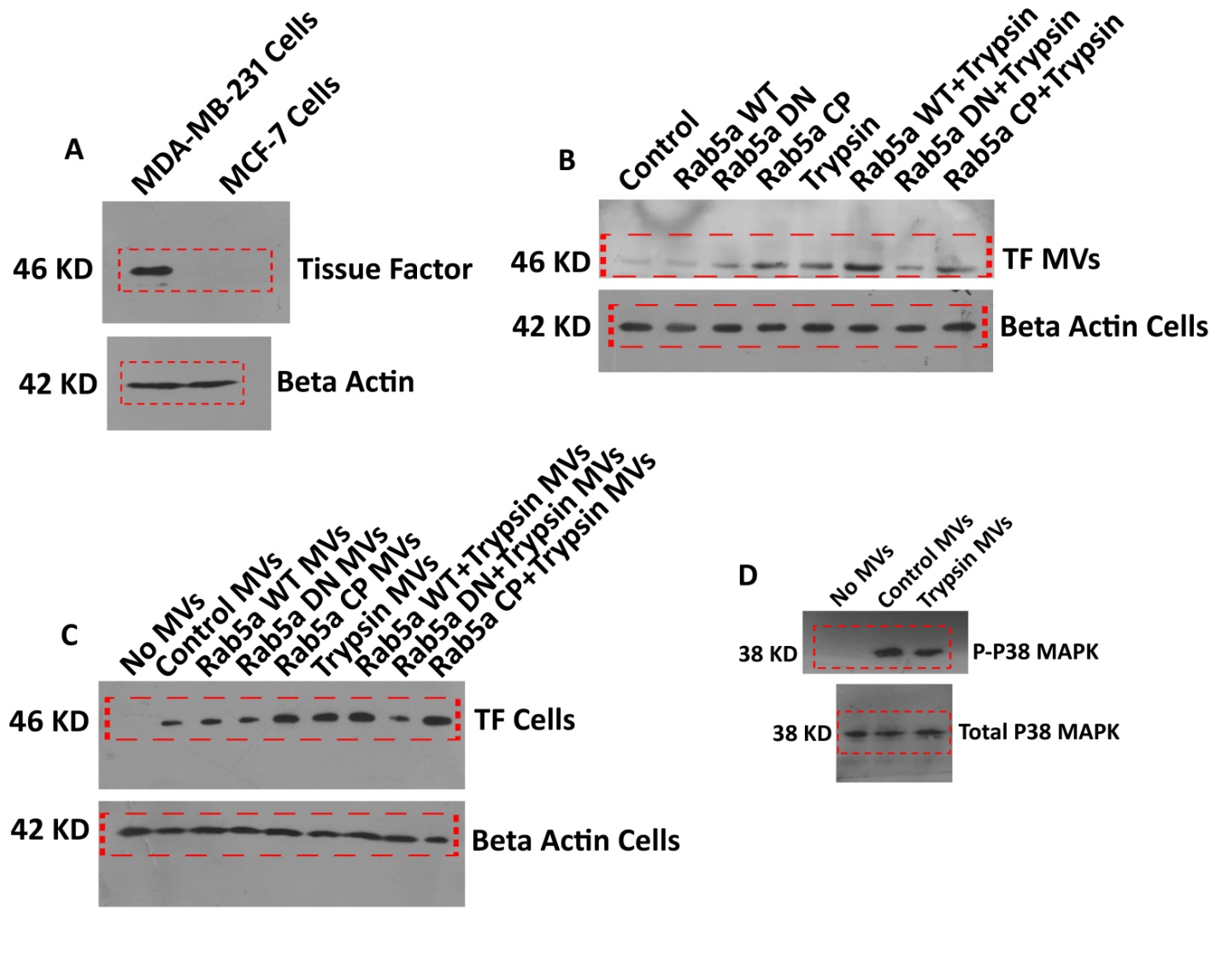


**Supplementary Figure S10.** Full length blots of Figure 6A (Represented here as A), Figure 6B (Represented here as B), Figure 6C (Represented here as C) and Figure 6H (Represented here as D). The red dotted lines indicate the cropping locations.

**Supplementary Figure S11**


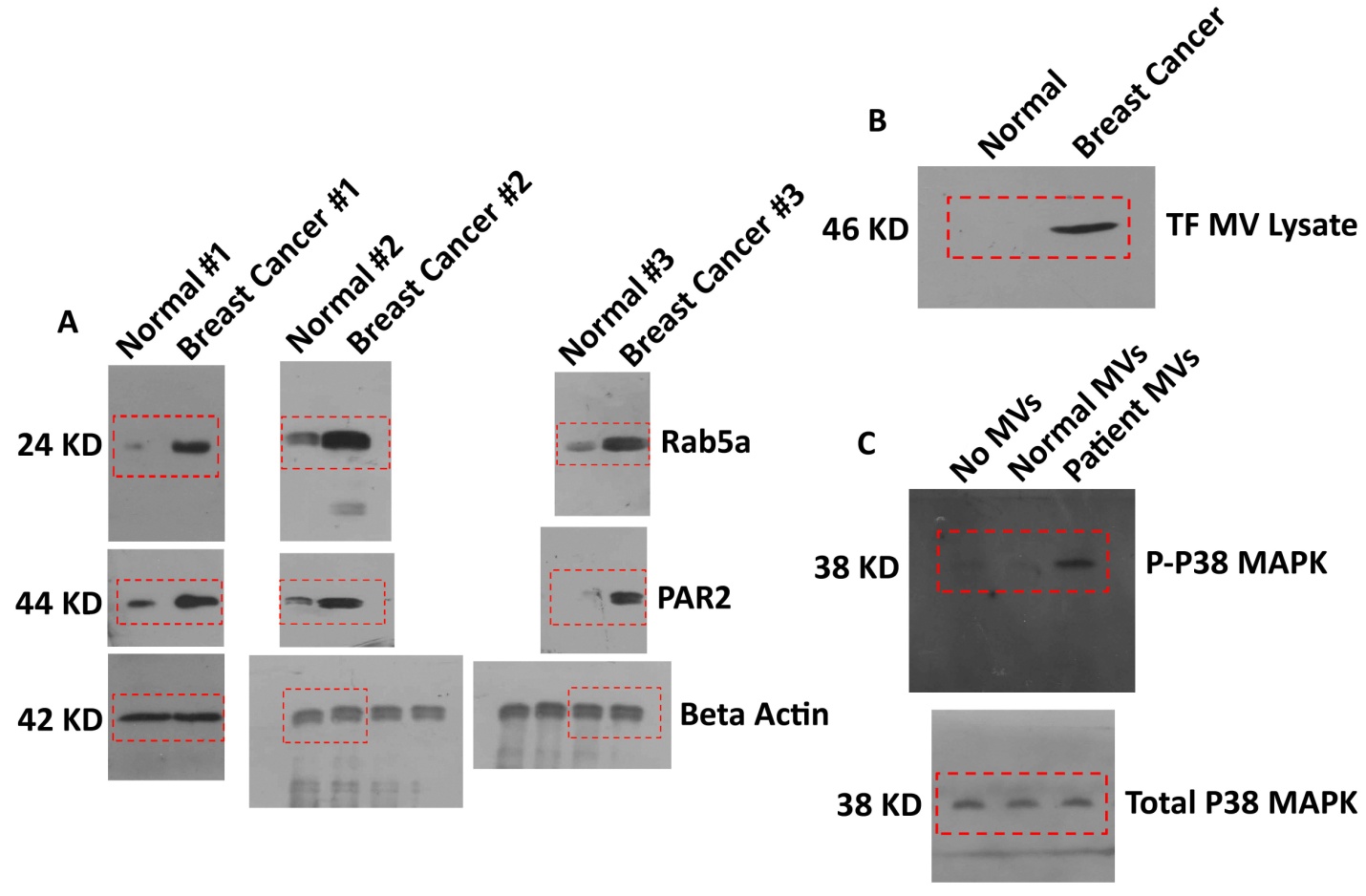


**Supplementary Figure S11.** Full length blots of Figure 7B (Represented here as A), Figure 7G (Represented here as B) and Figure 7M (Represented here as C). The red dotted lines indicate the cropping locations.

**Supplementary Figure S12**


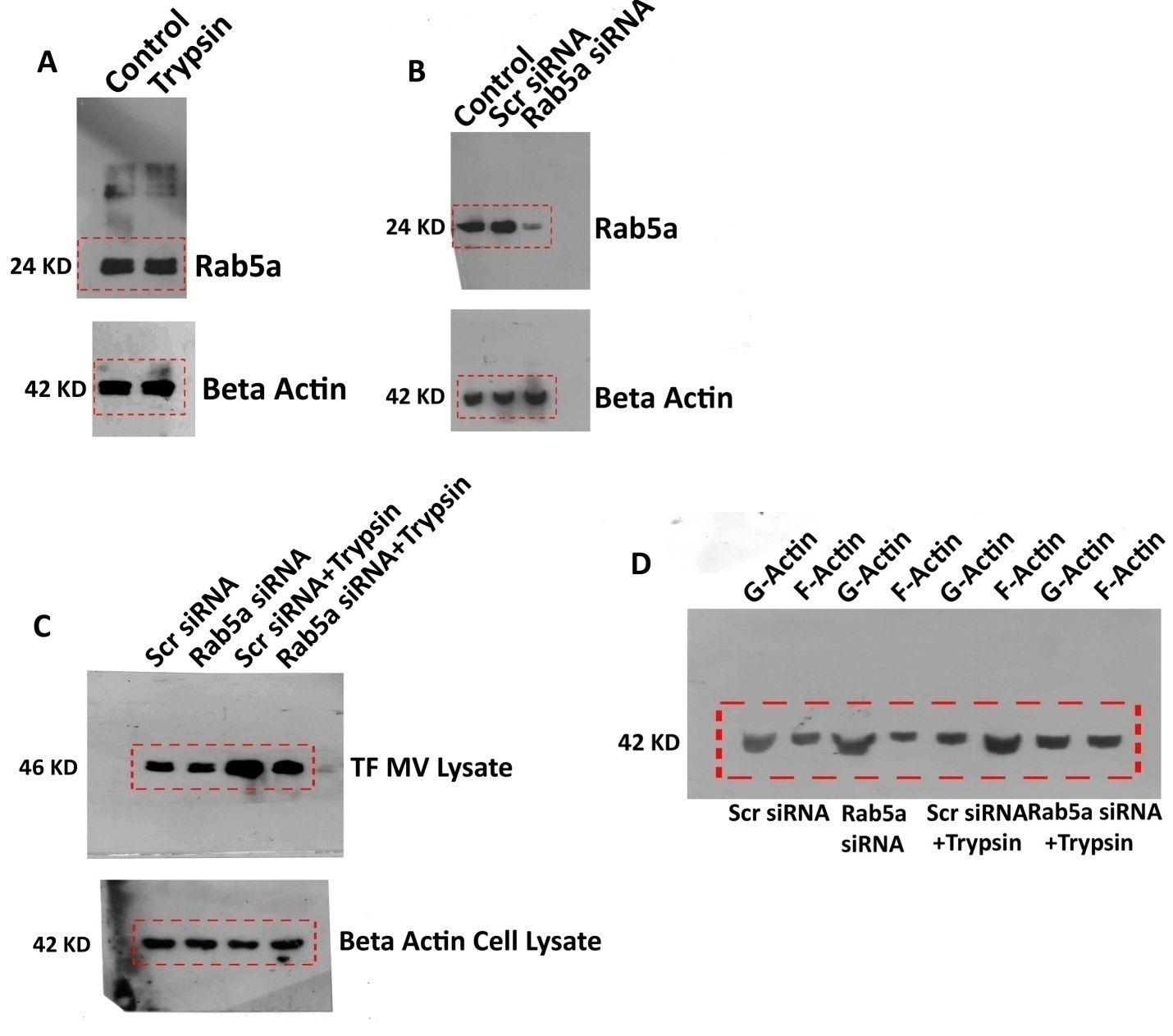


**Supplementary Figure S12.** Full length blots of Supplementary Figure S1A (Represented here as A), Supplementary Figure S1C (Represented here as B), Supplementary Figure S1D (Represented here as C) and Supplementary Figure S1F (Represented here as D). The red dotted lines indicate the cropping locations.

**Supplementary Figure S13**

**
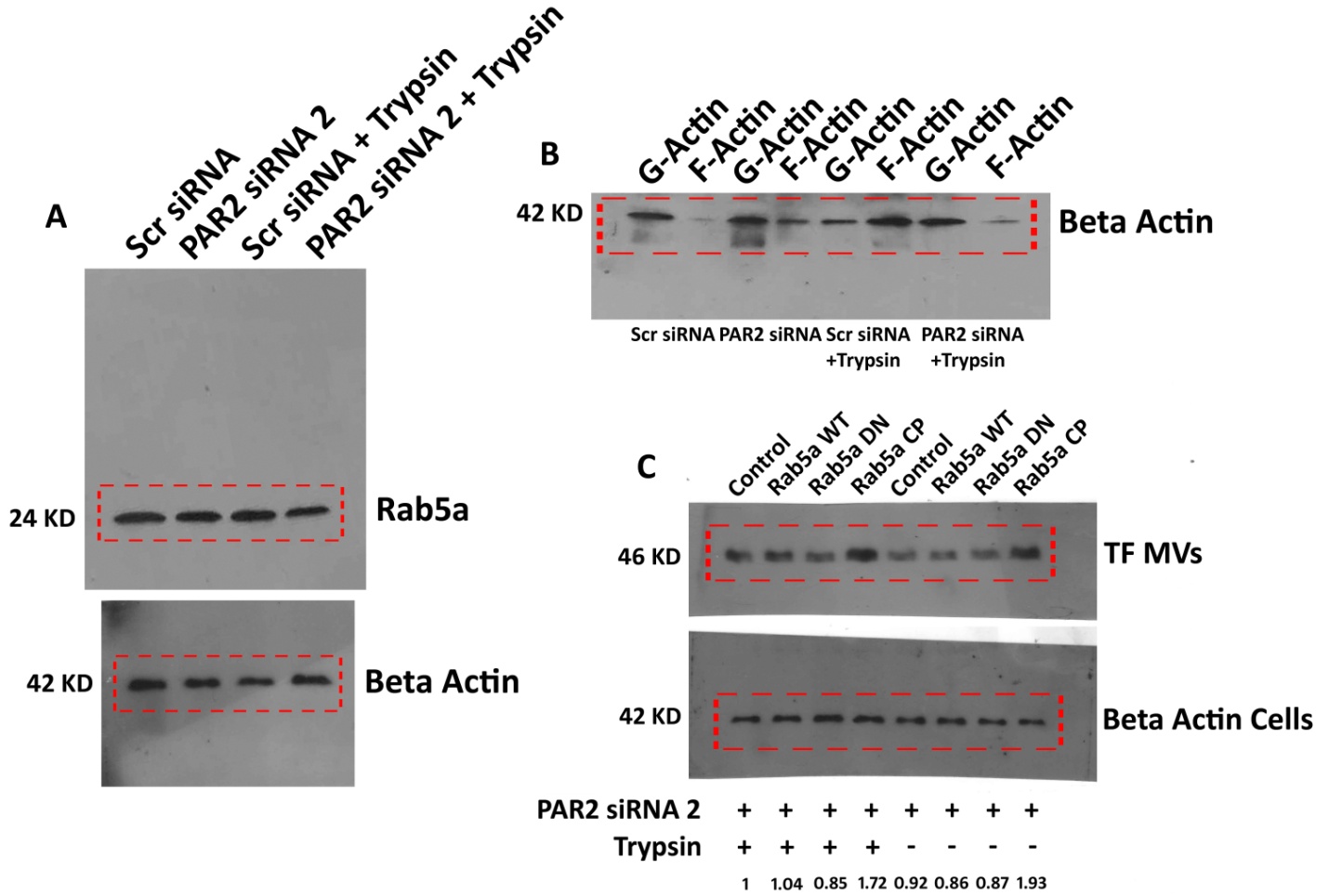
**

**Supplementary Figure S13.** Full length blots of Supplementary Figure S2A (Represented here as A), Supplementary Figure S2C (Represented here as B) and Supplementary Figure S2E (Represented here as C). The red dotted lines indicate the cropping locations.

**Supplementary Figure S14**


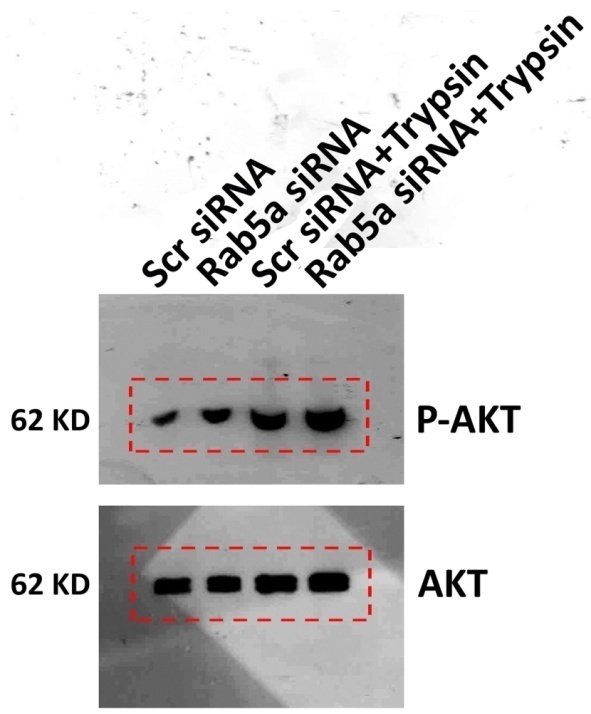


**Supplementary Figure S14.** Full length blots of Supplementary Figure S3A. The red dotted lines indicate the cropping locations.

**Supplementary Figure S15**


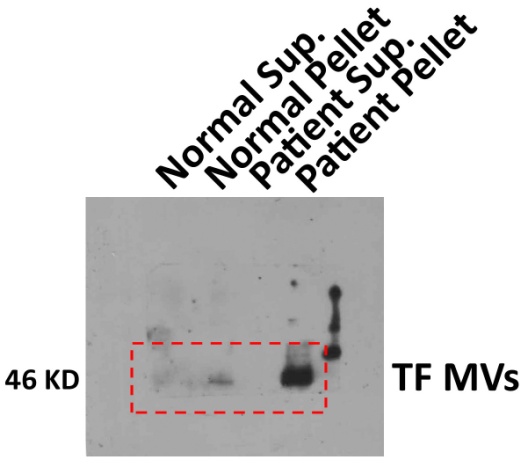


**Supplementary Figure S15.** Full length blot of Supplementary Figure S5A. The red dotted line indicates the cropping location.
